# Novel oral compound Z526 mitigates cancer-associated cachexia via intervening NF-κB signaling and oxidative stress

**DOI:** 10.1016/j.gendis.2024.101292

**Published:** 2024-04-08

**Authors:** Xiaofan Gu, Shanshan Lu, Shuang Xu, Yiwei Li, Meng Fan, Guangyu Lin, Yiyuan Liu, Yun Zhao, Weili Zhao, Xuan Liu, Xiaochun Dong, Xiongwen Zhang

**Affiliations:** a Shanghai Engineering Research Center of Molecular Therapeutics and New Drug Development, School of Chemistry and Molecular Engineering, East China Normal University, Shanghai 200062, China; b Department of Medicinal Chemistry, School of Pharmacy, Fudan University, Shanghai 201210, China; c Institute of Interdisciplinary Integrative Medicine Research, Shanghai University of Traditional Chinese Medicine, Shanghai 201203, China

**Keywords:** Cancer-associated cachexia, Fat lipolysis, Muscle atrophy, NF-κB signaling, Oxidative stress, Z526

## Abstract

Cancer-associated cachexia (CAC) is a severe metabolic disorder syndrome mainly characterized by muscle and fat loss, which accounts for one-third of cancer-related deaths. No effective therapeutic approach that could fully reverse CAC is available. NF-κB signaling and oxidative stress play vital roles in both muscle atrophy and fat loss in CAC. Here, we showed that our developed oral compound Z526 exhibited potent anti-CAC efficacy by inhibiting NF-κB signaling and ameliorating oxidative stress. *In vitro*, Z526 alleviated C2C12 myotube atrophy and 3T3-L1 adipocyte lipolysis induced by conditioned mediums of multiple cachectic tumor cells or pro-cachectic inflammatory cytokines. *In vivo*, Z526 attenuated the cachectic symptoms of C26 or LLC tumor-bearing mice. Z526 treatment reduced weight loss without impacting tumor growth and improved muscle atrophy, fat loss, and impaired grip force. Besides, serum TNF-α and IL-6 levels were reduced after Z526 treatment in C26 tumor-bearing mice. Of note, Z526 significantly prolonged the survival of LLC tumor-bearing mice. Activated NF-κB signaling and oxidative stress in cachectic muscle and fat tissues were reversed by Z526. Furthermore, Z526 exhibited a promising preclinical safety profile. Thus, oral Z526, which exhibited potent anti-CAC activities *in vitro* and *in vivo*, multiple interventions in diverse pathogenic mechanisms (NF-κB signaling and oxidative stress), and a favorable preclinical safety profile, holds the promise to be developed into a novel and beneficial therapeutic option for CAC.

## Introduction

Cancer-associated cachexia (CAC) occurs in patients with advanced cancers and leads to poor prognosis, reduced quality of life, and increased risk of death. CAC is a devastating syndrome of metabolic disorders characterized by weight loss (mainly of muscle and fat), anorexia, systematic inflammation, and redox imbalance.^1^^,^^2^ Data suggest that 50%–80% of cancer patients suffer from CAC, and CAC accounts for approximately 30% of cancer-related deaths.^3^ In the clinic, there are no potential therapeutic interventions that can completely reverse CAC. Anamorelin, a ghrelin receptor agonist and currently the only drug specifically targeting cachexia, was approved for marketing in Japan in December 2020.^4^^,^^5^ However, its clinical efficacy and improvement in quality of life have yet to be proven.

Due to the complex pathological mechanisms of CAC, single intervention therapeutic strategies, such as megestrol (an appetite simulant), Xilonix (an inhibitor of interleukin (IL)-1), and Enbosarm (a growth receptor mediator),^6^, ^7^, ^8^ have not achieved the expected results in clinical studies. Therefore, multiple interventions targeting different pathogenic mechanisms might be necessary for the treatment of CAC. NF-κB (nuclear factor-kappa B) signaling plays a crucial role in the onset and progression of CAC.^9^^,^^10^ It has been shown that NF-κB signaling is involved in both physiology (differentiation, growth, and metabolism) and pathology (cachexia, atrophy, and dystrophy) aspects of skeletal muscle.^11^ Inhibition of NF-κB in human fat cells could reduce tumor necrosis factor α (TNF-α)-mediated lipolysis, suggesting that NF-κB is essential for lipolysis.^12^ Also, oxidative stress, especially reactive oxygen species (ROS), is a key regulator in cachexia which could lead to mitochondrial dysfunction, increased activity of the ubiquitin-proteasome system, increased apoptosis, decreased protein synthesis, and dysregulated autophagy.^13^ In patients with CAC, increased ROS and decreased antioxidant levels in serum, as well as protein oxidation in skeletal muscle, have been observed, suggesting that ROS might be closely associated with cachexia.^14^^,^^15^ ROS production in adipocytes promoted lipolysis, which could be attenuated by N-acetylcysteine (an antioxidant).^16^ In all, NF-κB signaling and oxidative stress play key roles in the onset and progression of CAC, especially in muscle and fat loss, and therefore might offer therapeutic options for CAC.

Dithiocarbamates have been reported to exhibit a wide range of biological activities, including anti-inflammation, anti-oxidation, and anti-tumor,^17^^,^^18^ which makes it possible to combat CAC. Based on the skeleton of dithiocarbamates, we have designed and synthesized a novel series of compounds. Furthermore, we carried out drug screening from the series of compounds on the C2C12 myotube atrophy model of CAC and screened out a promising drug candidate Z526 with independent intellectual property rights (CN Patent No. ZL202011100806.X). Here, we have systematically evaluated the anti-CAC efficacy of the novel candidate compound (Z526) in multiple *in vitro* and *in vivo* models and explored Z526's pharmacological mechanisms of multiple interventions (NF-κB signaling and oxidative stress). Besides, the preclinical safety profile of Z256 was assessed.

## Materials and methods

### Cell culture

The associated information of cell lines, including C26 murine colon adenocarcinoma cells, MC38 murine colon adenocarcinoma cells, Lewis lung carcinoma (LLC) murine lung adenocarcinoma cells, HT-1080 human fibrosarcoma cells, PANC-1 human pancreatic adenocarcinoma cells, MIA PaCa-2 human pancreatic adenocarcinoma cells, is provided in Table S2.

### Cell models of cancer cachexia

Cell models of cancer cachexia were established as described in our previous reports.^19^ As the confluence of tumor cells (C26, LLC, HT-1080, PANC-1, and MIA PaCa-2) reached 80%, the medium was replaced with the fresh culture medium, followed by culture for 48 h. Then, the conditioned medium (CM) was harvested through centrifugation (at 5000 *g* at 4 °C for 10 min) and used to induce C2C12 myotube atrophy and mature 3T3-L1 adipocyte lipolysis subsequently.

During myotube differentiation, C2C12 myoblasts were seeded into plates and changed into a differentiation medium (Dulbecco's modified Eagle medium containing 2% horse serum) at 50% confluence. Fresh differentiation medium was changed every two days and myotube formation was observed after 5–6 days. To evaluate the efficacy of Z526 (purity >98%) against muscle atrophy *in vitro*, the CMs of tumor cells (mixed with the culture medium at a 1:1 ratio) or 100 ng/mL of recombinant cytokines (TNF-α or IL-6) were added to the medium that induced C2C12 myotube atrophy, along with different concentrations of Z526 for 48 h. The CM of non-tumor cells (such as 3T3-L1) was used as the control medium.

During adipocyte differentiation, 3T3-L1 adipocytes were seeded into plates and changed into differentiation medium I [culture medium containing 10 μg/mL of insulin (Solarbio, Beijing, China), 1 μM of dexamethasone (Sigma–Aldrich, St. Louis, MO, US), and 0.5 mM of 3-isobutyl-1methylxanthine (Sigma–Aldrich, St. Louis, MO, US)] at 100% confluence. 3T3-L1 adipocytes were incubated in differentiation medium I for 6 days, and the fresh differentiation medium I was changed every 3 days. After 6 days, the differentiation medium I was changed into differentiation medium II (culture medium containing 10 μg/mL of insulin) to induce adipocytes. After differentiation, lipid droplet formation was observed. To evaluate the efficacy of Z526 against lipolysis *in vitro*, the CMs of tumor cells (fixed with the culture medium at a 1:1 ratio) or 100 ng/mL of recombinant cytokines (TNF-α or IL-6) were added to induce mature 3T3-L1 adipocyte lipolysis, and simultaneously, various concentrations of Z526 were separately added, followed by incubation for 48 h. The CM of non-tumor cells (such as C2C12) was used as the control medium.

### Hematoxylin-eosin staining and determination of myotube diameters

C2C12 myotubes were fixed with 4% paraformaldehyde at room temperature for 1 h. After three washes with cold phosphate-buffered saline, cells were stained with hematoxylin and eosin by the standard procedure. Dissected muscle and adipose tissues were embedded in paraffin, cut into 10-μm-thickness slices, and stained with hematoxylin and eosin. The stained samples were observed and photographed with an optical microscope. Diameters of stained C2C12 myotubes and cross-sectional areas of myofibers in tissue slices were quantified through the software Image J.

### MTT assay

C2C12 myotubes and 3T3-L1 adipocytes were incubated with Z526 at various concentrations for 48 h, and then cell viability was measured by 3-(4,5-dimethylthiazol-2-γl)-2,5-diphenyltetrazolium bromide (MTT) assay.

### Oil red O staining and determination of lipid content

Fix 3T3-L1 adipocytes with 4% paraformaldehyde at room temperature for 1 h. To be stained, cells were incubated in the work solution (0.5% dye dissolved in 60% isopropanol) of oil red O (Sigma Aldrich, St. Louis, Mo, US) at room temperature for 30 min. The stained cells were observed and photographed with an optical microscope.

### Determination of glycerol and triglycerides

The levels of triglycerides in 3T3-L1 mature adipocytes and mouse serum were determined with a triglyceride quantification kit (Applygen, Beijing, China). The content of glycerol in the medium of 3T3-L1 mature adipocytes and mouse serum was detected with a lipolysis assay kit (Applygen, Beijing, P. R. China). All the operations above followed the instructions provided by the manufacturers.

### Western blotting assay

As described previously,^20^ a western blotting assay was performed to detect target protein levels in cells (C2C12 myotubes, 3T3-L1 adipocytes) and dissected tissues (muscle and fat). The used antibodies are listed in Table S3.

### Animals

Male BALB/c mice and C57bl/6j mice (6–8 weeks old) were purchased from Shanghai JH Laboratory Animal, Co., Ltd (Shanghai, China). Mice were housed on a 12:12 light–dark cycle in a temperature-controlled (21–23 °C) and specific pathogen-free environment and had free access to standard chow and water. All the animal care and operations abided by the Institutional Animal Care and Use Committee (IACUC) guidelines of East China Normal University.

### Animal models of cancer cachexia *in vivo*

To establish the C26 and LLC tumor-bearing mouse models of cancer cachexia, male BALB/c or C57bl/6j mice with the same initial body weight were divided into the healthy control group, the model group, and the treatment group. Mice in the model group and the treatment group were subcutaneously inoculated with 2 × 10^6^ of C26 colon adenocarcinoma cells or 3 × 10^6^ of LLC lung carcinoma cells in 100 μL of sterile phosphate-buffered saline into the right flank of mice, respectively. Mice in the healthy control group and the model group were administered with a vehicle while mice in the Z526 treatment group were administered with Z526 daily. To compare the anti-CAC efficacy of Z526 with that of anamorelin, the anamorelin treatment group with oral administration of anamorelin (30 mg/kg) every day was also included in the study. The cachexia-associated parameters were daily monitored and recorded on the day of inoculation, including tumor volume (calculation formula: tumor volume = X^2^ × Y × 0.5; X is the shortest diameter of tumor, and Y is the longest diameter of tumor), body weight (with/without tumors), body temperature, food intake, survival rate, *etc*. Mice were euthanized and the tissues including tumors, serum, gastrocnemius muscle (GA), and epididymal white fat (eWAT) were dissected and weighed immediately. The tissues were rapidly frozen in liquid nitrogen and then stored at −80 °C for further study.

### Enzyme-linked immunosorbent assay

TNF-α and IL-6 levels in mouse serum were determined by their enzyme-linked immunosorbent assay kits, following the instructions provided by the manufacturers. Absorbance was measured by SpectraMax M5 microplate reader (Molecular Device, USA).

### Immunofluorescent staining assay

To evaluate the translocation of p65 into the nucleus in C2C12 myotubes and 3T3-L1 adipocytes, an immunofluorescent staining assay was conducted, as previously described.^21^ After treatment, the cells were fixed with 4% paraformaldehyde for 30 min, permeabilized with 0.2% Triton X-100 for 30 min, and then blocked with 5% bovine serum albumin at room temperature for 1 h. Cells were incubated with diluted anti-p65 antibody and anti-β-actin antibody at 4 °C overnight. The next day, cells were incubated with fluorescence-conjugated secondary antibodies for 2 h and counterstained with DAPI for 10 min at temperature. The images were captured by Cytation 5 image reader (BioTek, USA) and quantified by Image J software.

### NF-κB luciferase reporter assay

NF-κB's transcriptional activity was determined using NF-κB luciferase reporter assay as previously described.^22^ HEK293T cells carrying the NF-κB-responsive luciferase reporter constructs, which were obtained from BoyaoBio (Guangzhou, China), were seeded in 96-well microplates and treated with CMs of cachectic tumor cells or TNF-α in the presence or absence of Z526 for 48 h. According to the manufacturer's instructions, luciferase activity was measured by a dual-luciferase reporter assay system (Promega) using a SpectraMax M5 microplate luminometer (Molecular Device, USA).

### ROS detection

ROS levels were detected in C2C12 myotubes and 3T3-L1 adipocytes with a ROS assay kit according to the manufacturer's instructions. In brief, cells were stained with 2′,7′-dichlorofluorescin diacetate (DCFH-DA) at room temperature for 30 min, after which the images were captured by fluorescent microscope (Olympus, Japan) and fluorescence signals were detected by SpectraMax M5 microplate fluorometer (Molecular Device, USA).

### Quantitative real-time PCR

To measure mRNA levels of NADPH oxidases in C2C12 myotubes and 3T3-L1 adipocytes and mRNA levels of zinc α2-glycoprotein 1 and hormone-sensitive lipase (HSL) in 3T3-L1 adipocytes, quantitative real-time PCR was performed with CFX96 Touch Deep Well Real-Time PCR System (Bio-rad, CA, USA) as previously described.^22^ The used primers were synthesized by Sangon Bio (shanghai, China), of which sequences are shown in Table S4.

### Maximum tolerated dose

ICR mice were orally administered with Z526 (25, 50, 100 mg/kg) once daily and a vehicle was used as the control. Body weight was monitored for 14 days.

### Histology analysis

Representative mice were sacrificed and their tissues were harvested and fixed in 4% paraformaldehyde at room temperature for 1 h. Then, fixed tissues were embedded in paraffin, cut into 10-μM-thickness sections, and stained with hematoxylin and eosin. The histology slices were used for quantitative analysis or pathological analysis of cross-sectional areas.

### hERG blockade assay

The effect of Z526 on the human ether-a-go-go related gene (hERG) potassium channel was determined using the whole-cell path clamp technique.^23^ The established hERG-stably-expressed CHO cells were intervened by Z526 at various concentrations to record the current changes of hERG potassium channels. The current of hERG potassium channels was recorded and analyzed through the software pCalmp10. The calculation of the inhibition rate was as follows: inhibition rate (%) = [1 − (I ÷ I0)] × 100% (inhibitory rate: Z526's inhibitory rate of hERG potassium channels' current; I: tail-peak current of hERG potassium channels in the absence of Z526; I0: tail-peak current of hERG potassium channels in the presence of Z526).

### Statistical analysis

Numerical data were expressed as mean ± standard error of the mean. Statistical analysis was performed using a two-tailed student's *t*-test or one-way ANOVA with GraphPad Prism 8.0 statistical program. A difference with *P* < 0.05 was considered statistically significant.

## Results

### Z526 alleviates C2C12 myotube atrophy induced by simulated cancer cachexia injuries

**Figure 1:**
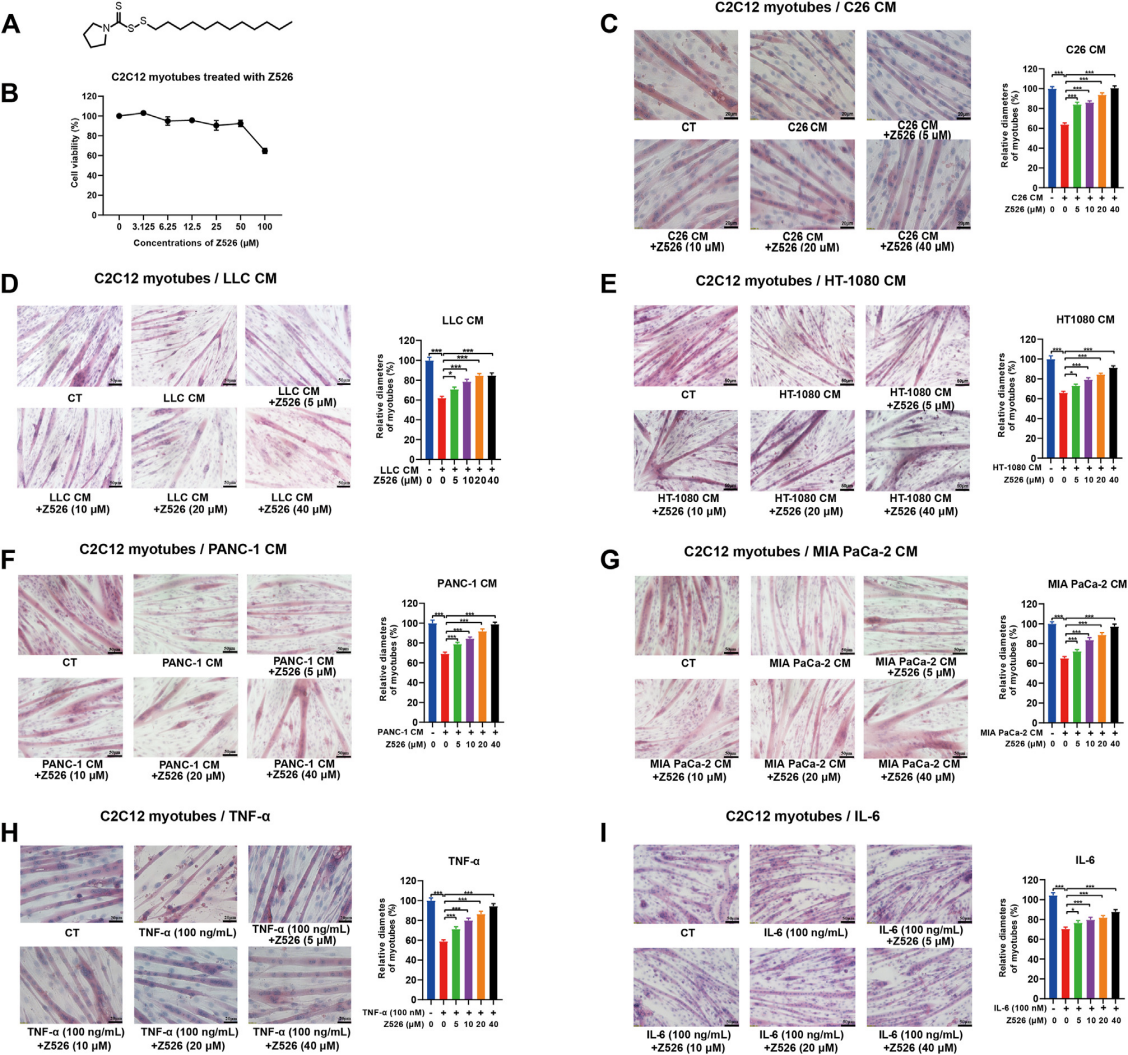
Z526's effects on C2C12 myotube atrophy induced by simulated cachectic injuries *in vitro*. C2C12 myotubes were treated with CMs of cachectic tumor cells or pro-cachectic inflammatory cytokines in the presence or absence of Z526 at various concentrations for 48 h. **(A)** The structural formula of Z526. **(B)** Z526's effects on cell viability of C2C12 myotubes. **(C–I)** Representative images and quantitative analysis results of diameters of C2C12 myotubes induced by (C) CM of C26 tumor cells, (D) CM of LLC tumor cells, (E) CM of HT-1080 tumor cells, (F) CM of PANC-1 tumor cells, (G) CM of MIA PaCa-2 tumor cells, (H) recombinant TNF-α, or (I) recombinant IL-6 respectively. Scale bar, 20 or 50 μm. The data represent mean ± standard error of the mean; ∗∗∗*P* < 0.001, ∗∗*P* < 0.01, ∗*P* < 0.05. CM, conditioned medium; IL-6, interleukin 6; TNF-α, tumor necrosis factor α.

The structural formula of Z526 is shown in Figure 1A. To determine whether Z526 could alleviate muscle atrophy, we used the murine C2C12 myotube atrophy model, a well-established cell model for CAC. As shown in Figure 1B, 40 μM of Z526 was non-toxic to C2C12 myotubes, identified by MTT assay. After differentiation, C2C12 myotubes were treated with the CM of various cachectic tumor cells, including C26, LLC, HT-1080, PANC-1, and MIA PaCa-2 cells, in the presence or absence of Z526. As expected, myotube diameters were reduced post-treatment of CMs, and Z526 significantly reversed this reduction in a dose-dependent manner (Fig. 1C–G). For instance, the diameters of C26 CM-induced myotubes, decreased to 63.94% of the control, while that of Z526-treated myotubes returned to 84.06%, 86.01%, 93.88%, and 100.69% of the control at various concentrations, respectively (Fig. 1C). As shown in Figure 1H and I, Z526, as well, dose-dependently ameliorated myotube atrophy induced by recombinant TNF-α or IL-6. Of note, Z526 did not affect the diameters of untreated myotubes or non-cachectic MC38 CM-induced myotubes (Fig. S1A, B). These results suggested that Z526 could alleviate C2C12 myotube atrophy induced by simulated cancer cachexia injuries.

### Z526 alleviates 3T3-L1 adipocyte lipolysis induced by simulated cancer cachexia injuries

**Figure 2:**
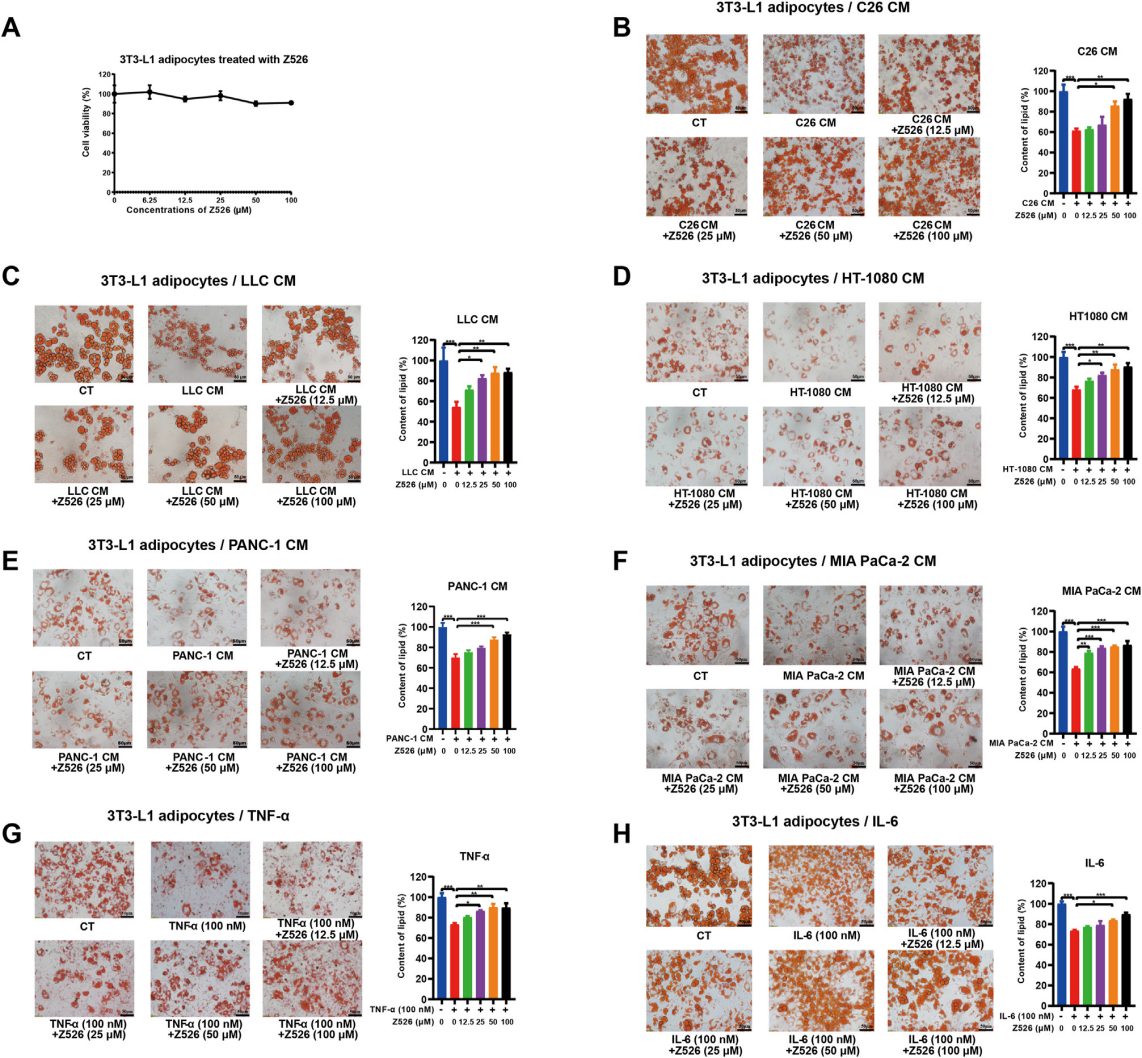
Z526's effects on 3T3-L1 adipocyte lipolysis induced by simulated cachectic injuries *in vitro*. 3T3-L1 adipocytes were treated with CMs of cachectic tumor cells or pro-cachectic inflammatory cytokines in the presence or absence of Z526 at various concentrations for 48 h. **(A)** Z526's effects on cell viability of 3T3-L1 adipocytes. **(B–H)** Representative images and quantitative analysis results of lipid content of 3T3-L1 adipocytes treated with (B) CM of C26 tumor cells, (C) CM of LLC tumor cells, (D) CM of HT-1080 tumor cells, (E) CM of PANC-1 tumor cells, (F) CM of MIA PaCa-2 tumor cells, (G) recombinant TNF-α, or (H) recombinant IL-6, respectively. Scale bar, 20 or 50 μm. The data represent mean ± standard error of the mean; ∗∗∗*P* < 0.001, ∗∗*P* < 0.01, ∗*P* < 0.05. CM, conditioned medium; IL-6, interleukin 6; TNF-α, tumor necrosis factor α.

To determine whether Z526 could alleviate lipolysis, we used the mature murine 3T3-L1 adipocyte lipolysis model, a common cell model for CAC. As shown in Figure 2A, 100 μM of Z526 was non-toxic to 3T3-L1 adipocytes, determined by MTT assay. The differentiated mature 3T3-L1 adipocytes were treated with the CM of multiple cachectic tumor cells, including C26, LLC, HT-1080, PANC-1, and MIA PaCa-2 cells, in the presence or absence of Z526. Lipid content in adipocytes was decreased by CMs, and Z526 significantly reversed the decrease in a dose-dependent manner (Fig. 2B–F). For example, the lipid content of C26 CM-induced adipocytes, dropped to 61.35% of the control, while that of Z526-treated adipocytes restored to 62.77%, 67.14%, 85.81%, and 92.34% of the control at various concentrations, respectively (Fig. 2B). As shown in Figure 2G and H, Z526, as well, dose-dependently ameliorated adipocyte lipolysis induced by recombinant TNF-α or IL-6. Correspondingly, Z526 also reversed an increase in released free glycerol and a decrease in intracellular triglyceride accumulation of 3T3-L1 adipocytes, treated with C26 CM, TNF-α, or IL-6 (Fig. S2). It is worth noting that Z526 did not affect the lipid content of untreated adipocytes or non-cachectic MC38 CM-induced adipocytes (Fig. S1C, D). These results demonstrated that Z526 ameliorated 3T3-L1 adipocyte lipolysis induced by simulated cancer cachexia injuries.

### Z526 exhibits anti-cachexia efficacy in tumor-bearing mice

To validate the anti-CAC effect of Z526 *in vivo* models, we established C26 and LLC tumor-bearing mouse models by subcutaneously implanting tumor cells into the right flanks of mice. The mice then were randomly divided into healthy control groups (without tumors), model groups (with tumors), and treatment groups (with tumors). Compared with healthy mice, tumor-bearing mice lost weight rapidly, while Z526 treatment could attenuate and delay the cachectic symptoms.

**Figure 3:**
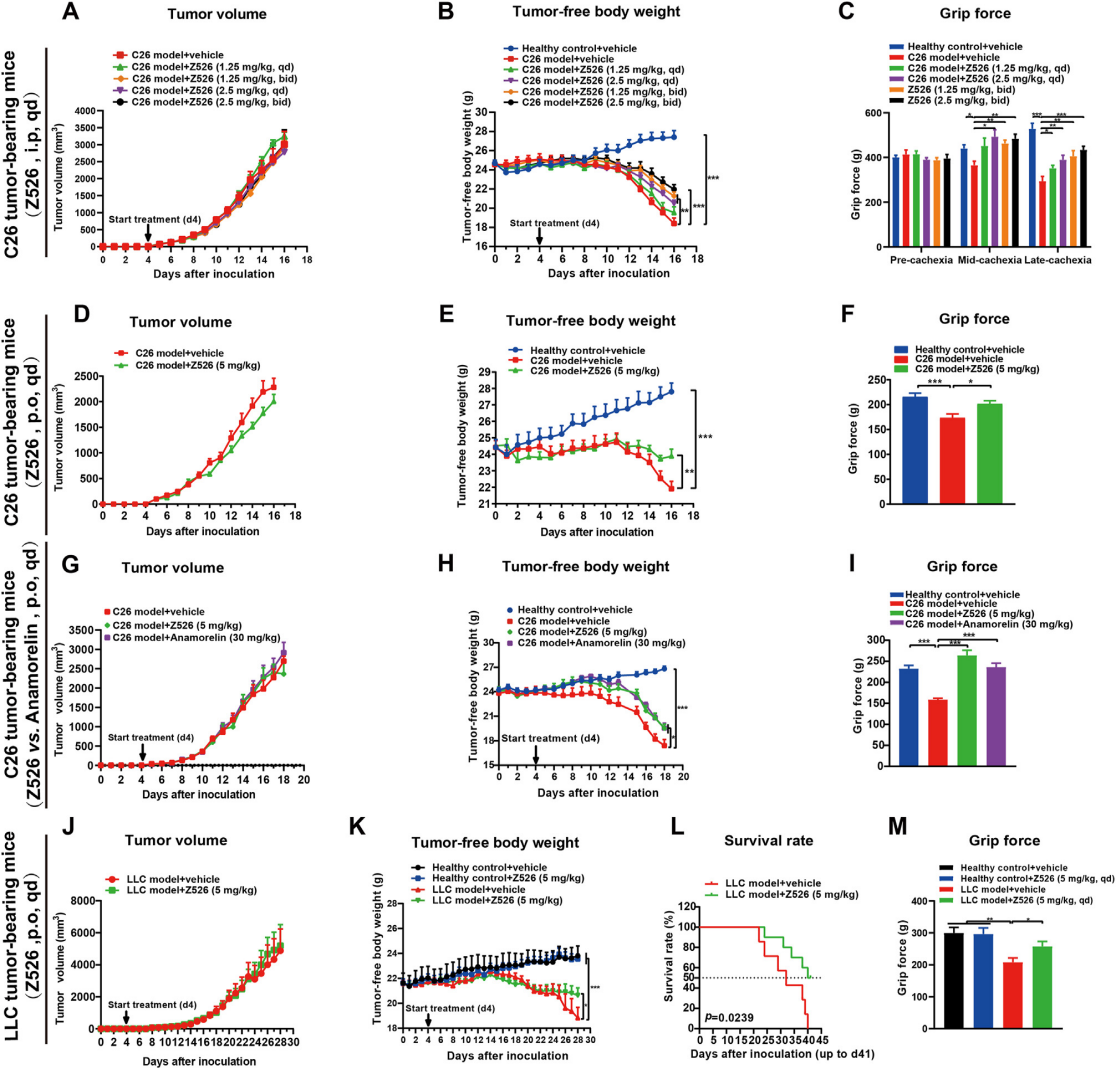
Z526 attenuates the cachectic symptoms of tumor-bearing mice *in vivo*. **(A**–**C)** C26 tumor-bearing mice were intraperitoneally administered with Z526 and monitored for (A) tumor volume, (B) tumor-free body weight, and (C) grip force. **(D**–**F)** C26 tumor-bearing mice were orally administered with Z526 and monitored for (D) tumor volume, (E) tumor-free body weight, and (F) grip force. **(G**–**I)** C26 tumor-bearing mice were orally administered Z526 or anamorelin and monitored for (G) tumor volume, (H) tumor-free body weight, and (I) grip force. **(J**–**M)** LLC tumor-bearing mice were orally administered and monitored for (J) tumor volume, (K) tumor-free body weight, (L) survival rate, and (M) grip force. The data represent mean ± standard error of the mean; *n* = 8; ∗∗∗*P* < 0.001, ∗∗*P* < 0.01, ∗*P* < 0.05.

In C26 tumor-bearing mice, intraperitoneal injection of Z526 could reduce weight loss in a dose-dependent manner without affecting tumor growth (Fig. 3A, B). Moreover, C26 tumor-bearing mice exhibited lower grip force than healthy mice, which could be improved by Z526 treatment (Fig. 3C).

To assess the effectiveness of oral Z526, 5 mg/kg of Z526 was given to C26 tumor-bearing mice through intragastric administration. The following results confirmed that Z526 was orally available for anti-CAC without the influence of C26 tumor growth (Fig. 3D–F; Fig. S3A, B). Z526 slightly improved the food intake and body temperature of C26 tumor-bearing mice (Fig. S3C, D).

For comparing the anti-CAC effects between Z526 and anamorelin, C26 tumor-bearing mice were orally administered with either Z526 or anamorelin. The results showed that Z526 at a dose of 5 mg/kg exhibited similar efficacy as anamorelin at a dose of 30 mg/kg (Fig. 3H; Fig. S3E, F). Notably, Z526 was found to restore the impaired grip force of C26 tumor-bearing mice, even better than anamorelin (Fig. 3I).

Additionally, Z526 was found to be able to ameliorate marked weight loss in LLC tumor-bearing mice without affecting tumor growth (Fig. 3J–M; Fig. S3I, J). Likely, oral treatment of Z526 could restore the impaired grip force of mice bearing LLC tumors (Fig. 3M). Of note, oral Z526 treatment significantly prolonged the survival time of LLC tumor-bearing mice (Fig. 3L). Likely, Z526 slightly improved the food intake and body temperature of LLC tumor-bearing mice (Fig. S3K, L). Collectively, these findings supply evidence that Z526 could attenuate cachectic symptoms of various tumor-bearing mouse CAC models.

### Z526 reduces muscle and fat loss in C26 tumor-bearing mice

**Figure 4:**
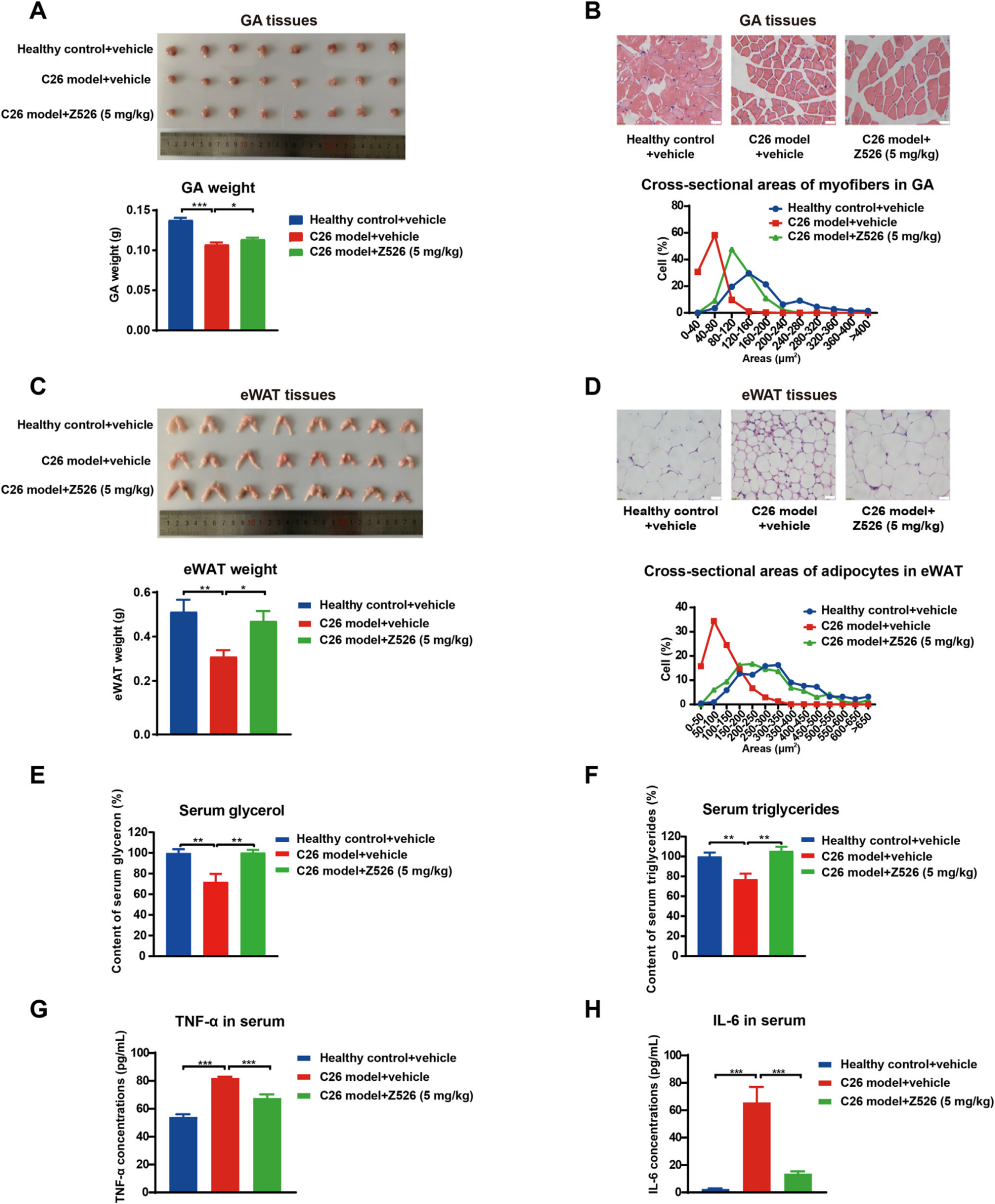
Z526 reduces loss of muscle and fat and inflammatory cytokines in C26 tumor-bearing mice. At the endpoint of the experiment, C26 tumor-bearing mice were executed and tissues of interest were dissected for further analysis. **(A)** Representative images of GA and weight analysis and **(B)** CSA of myofibers in GA tissues and quantitative statistics. **(C)** Representative images of eWAT and weight analysis and **(D)** CSA of adipocytes in eWAT tissues and quantitative statistics. **(E)** Glycerol and **(F)** triglyceride levels in mice serum. **(G)** TNF-α levels and **(H)** IL-6 levels in mice serum. The data represent mean ± standard error of the mean; *n* = 8; ∗∗∗*P* < 0.001, ∗∗*P* < 0.01, ∗*P* < 0.05. CSA, cross-sectional area; eWAT, epididymal white fat; GA, gastrocnemius muscle; IL-6, interleukin 6; TNF-α, tumor necrosis factor α.

Since muscle and fat loss are the two main distinct features of CAC, we checked whether Z526 could reduce muscle and fat wasting in the C26 tumor-bearing mouse model of CAC. After 16 days of Z526's oral treatment, C26 tumor-bearing mice were executed to dissect tissues of interest. C26 tumor burden led to a remarkable reduction of GA and eWAT weight, which could be significantly reversed by Z526 treatment (Fig. 4A–C). The cross-sectional area of GA myofibers was much smaller in C26 tumor-bearing mice than in healthy mice, while Z526 treatment could ameliorate this reduction (Fig. 4B). Similarly, Z526 attenuated the decrease of eWAT adipocytes' cross-sectional area (Fig. 4D). Z526 treatment significantly alleviated the decrease of glycerol and triglycerides in the serum of C26 tumor-bearing mice (Fig. 4E, F). In addition, elevated TNF-α and IL-6 levels in the serum of C26 tumor-bearing mice were observed, which could be down-regulated to near-normal levels by Z526 (Fig. 4G, H). These findings provided sufficient evidence that Z526 could reduce cachectic muscle and fat loss.

### Z526 inhibits NF-κB signaling in cachectic muscle and fat

**Figure 5:**
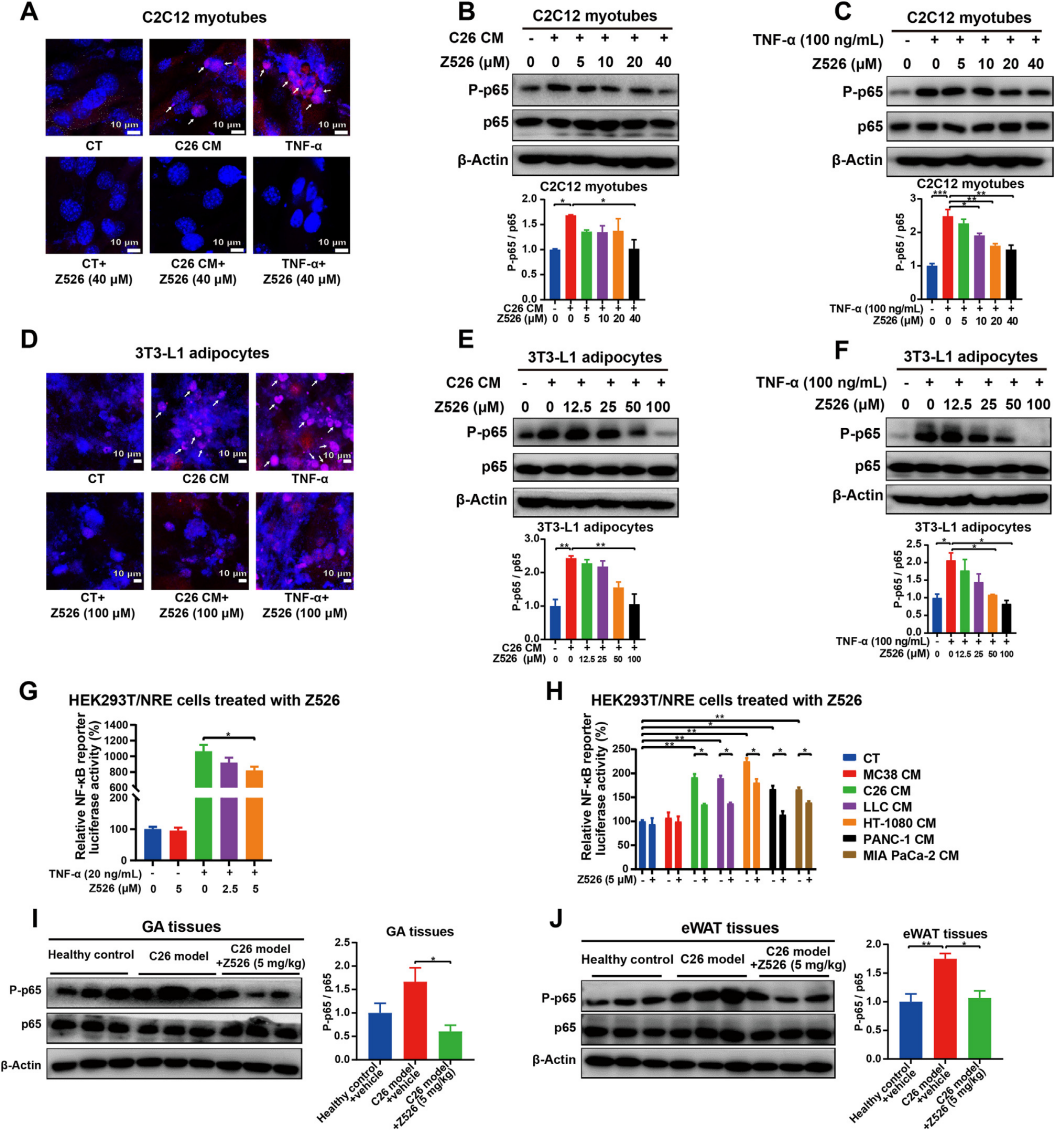
Z526 inhibits nuclear translocation, phosphorylation and transcription activity of NF-κB in the cachectic setting. C2C12 myotubes and 3T3-L1 adipocytes were incubated with C26 CM or TNF-α in the presence or absence of Z526. **(A)** Representative florescent images of myotubes stained with antibodies against p65 (red) and counterstained with DAPI (blue). **(B, C)** Western blotting analysis for phosphorylation levels of p65 in myotubes. **(D)** Representative florescent images of adipocytes stained with antibodies against p65 (red) and counterstained with DAPI (blue). **(E, F)** Western blotting analysis for phosphorylation levels of p65 in adipocytes. **(G, H)** Transcription activities of NF-κB induced by TNF-α or CMs of multiple cachectic tumor cells, in the presence or absence of Z526. **(I)** Western blotting analysis for phosphorylation levels of p65 in GA tissues of C26 tumor-bearing mice. **(J)** Western blotting analysis for phosphorylation levels of p65 in eWAT tissues of C26 tumor-bearing mice. Scale bar, 10 μm. Arrows indicate nuclear localization of NF-κB. The data represent the mean ± SEM, ^∗∗∗^*P* < 0.001, ^∗∗^*P* < 0.01, ^∗^*P* < 0.05. CM, conditioned medium; NF-κB, nuclear factor-kappa B; TNF-α, tumor necrosis factor α; GA, gastrocnemius muscle; eWAT, epididymal white fat.

As active NF-κB is phosphorylated and translocated into nuclei to perform its function as a transcription factor, we investigated whether Z526 could affect the activation of NF-κB by evaluating the nuclear translocation, phosphorylation levels, and transcriptional activity of NF-κB in cachectic muscle and fat. To achieve this, C2C12 myotubes or 3T3-L1 adipocytes were treated with C26 CM or TNF-α in the presence or absence of Z526, after which NF-κB location was determined by a confocal microscope. It was found that NF-κB was localized within the nucleus following induction with C26 CM or TNF-α, which was inhibited by Z526 (Fig. 5A–D). Interestingly, C26 CM or TNF-α-induced up-regulation of p65 phosphorylation was significantly reduced by Z526 in C2C12 myotubes and 3T3-L1 adipocytes (Fig. 5B, C, E, F).

HEK-293T cells containing NF-κB luciferase reporter constructs were used to assess NF-κB transcriptional activity. The results showed that Z526 diminished NF-κB transcriptional activity, which was enhanced by TNF-α or CMs of cachectic tumor cells (Fig. 5G, H).

Consistently, elevated p65 phosphorylation in muscle and fat tissues dissected from C26 tumor-bearing mice could notably be reversed after Z526 treatment (Fig. 5I, J). These results above demonstrated that Z526 inhibited NF-κB signaling in cachectic muscle and fat.

### Z526 reduces ROS levels in cachectic muscle and fat

**Figure 6:**
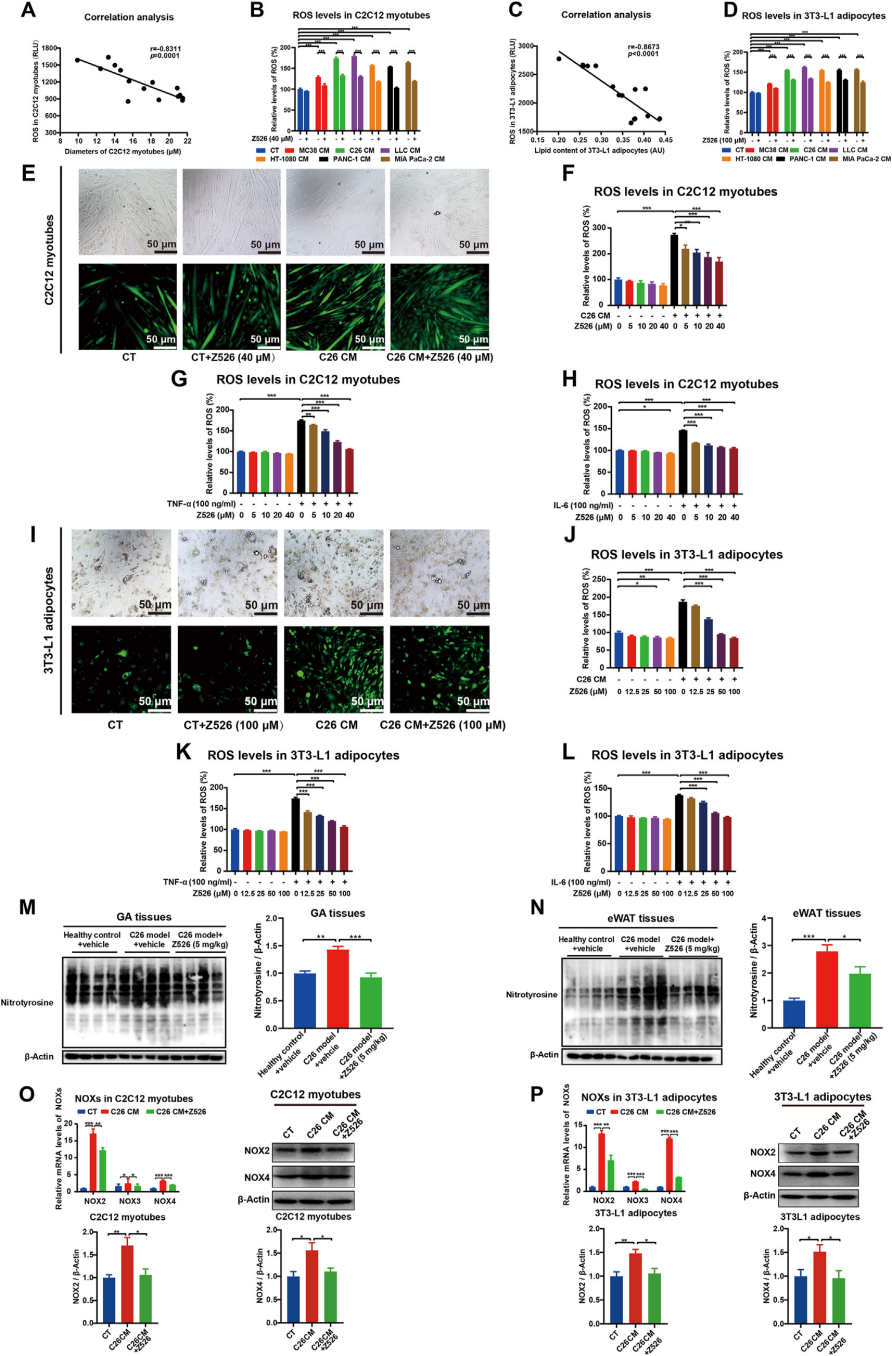
Z526 reduces ROS levels in cachectic muscle and fat. C2C12 myotubes and 3T3-L1 adipocytes were incubated with CMs of tumor cells or pro-cachectic inflammatory cytokines in the presence or absence of Z526. DCFH-DA (2′,7′-dichlorofluorescin diacetate) was used as a fluorescent probe to detect ROS levels. **(A)** Correlation analysis between myotube diameters and ROS levels in C2C12 myotubes. **(B)** ROS levels in C2C12 myotubes treated with CMs in the presence or absence of Z526. **(C)** Correlation analysis of lipid content and ROS levels in 3T3-L1 adipocytes. **(D)** ROS levels in 3T3-L1 adipocytes treated with CMs in the presence or absence of Z526. **(E)** Representative fluorescent images (scale bar, 50 μm) of C2C12 myotubes treated with C26 CM in the presence or absence of Z526, demonstrating ROS levels. **(F–H)** Quantitative analysis of ROS in C2C12 myotubes treated with C26 CM, TNF-α, or IL-6 in the presence or absence of Z526. **(I)** Representative fluorescent images (scale bar = 50 μm) of 3T3-L1 adipocytes treated with C26 CM in the presence or absence of Z526, demonstrating ROS levels. **(J**–**L)** Quantitative analysis of ROS in 3T3-L1 adipocytes incubated with C26 CM, TNF-α, or IL-6 in the presence or absence of Z526. After oral treatment of Z526, GA, and eWAT tissues were dissected from executed C26 tumor-bearing mice. **(M, N)** Western blotting analysis for nitrotyrosine levels in GA and eWAT tissues of C26 tumor-bearing mice. **(O, P)** Relative mRNA and protein levels of NADPH oxidases (NOXs) in C2C12 myotubes and 3T3-L1 adipocytes treated with C26 CM in the presence or absence of Z526. The data represent mean ± standard error of the mean; ∗∗∗*P* < 0.001, ∗∗*P* < 0.01, ∗*P* < 0.05. CM, conditioned medium; eWAT, epididymal white fat; GA, gastrocnemius muscle; IL-6, interleukin 6; ROS, reactive oxygen species; TNF-α, tumor necrosis factor α.

Through testing ROS levels in C2C12 myotubes induced by CMs of cachectic tumor cells (Fig. 1), we observed that ROS levels had a negative correlation with myotube diameters (Fig. 6A). Similarly, there was also a negative correlation between ROS levels and cellular lipid content in cachectic tumor cells' CM-induced 3T3-L1 adipocytes (Figure 2, Figure 6C). As expected, ROS levels in both C2C12 myotubes and 3T3-L1 adipocytes were remarkably elevated by CMs of cachectic tumor cells (C26, LLC, HT-1080, PANC-1, MIA PaCa-2), compared with non-cachectic tumor cell (MC38), which could be reversed by Z526 (Fig. 6B–D). Furthermore, the increased ROS levels in C2C12 myotubes treated with C26 CM or cachectic pro-inflammatory cytokines (TNF-α, IL-6) were observed to be abolished dose-dependently by Z526 treatment (Fig. 6F, G). Similar results were found in 3T3-L1 adipocytes (Fig. 6J–L). Representative fluorescence images are presented in Figure 6E–I.

Compared with the healthy mice, both muscle and fat tissues from C26 tumor-bearing mice demonstrated intensified protein nitrosylation levels but significantly reduced after Z526 treatment (Fig. 6M, N). Furthermore, we observed a significant increase in mRNA and protein expression of NADPH oxidases of C26 CM-induced myotubes and adipocytes, which were reversed by Z526 treatment (Fig. 6O, P). These findings indicated that Z526 reduced the excessive ROS levels in cachectic muscle and fat.

### Z526 regulates metabolic signaling pathways mediated by NF-κB or ROS

**Figure 7:**
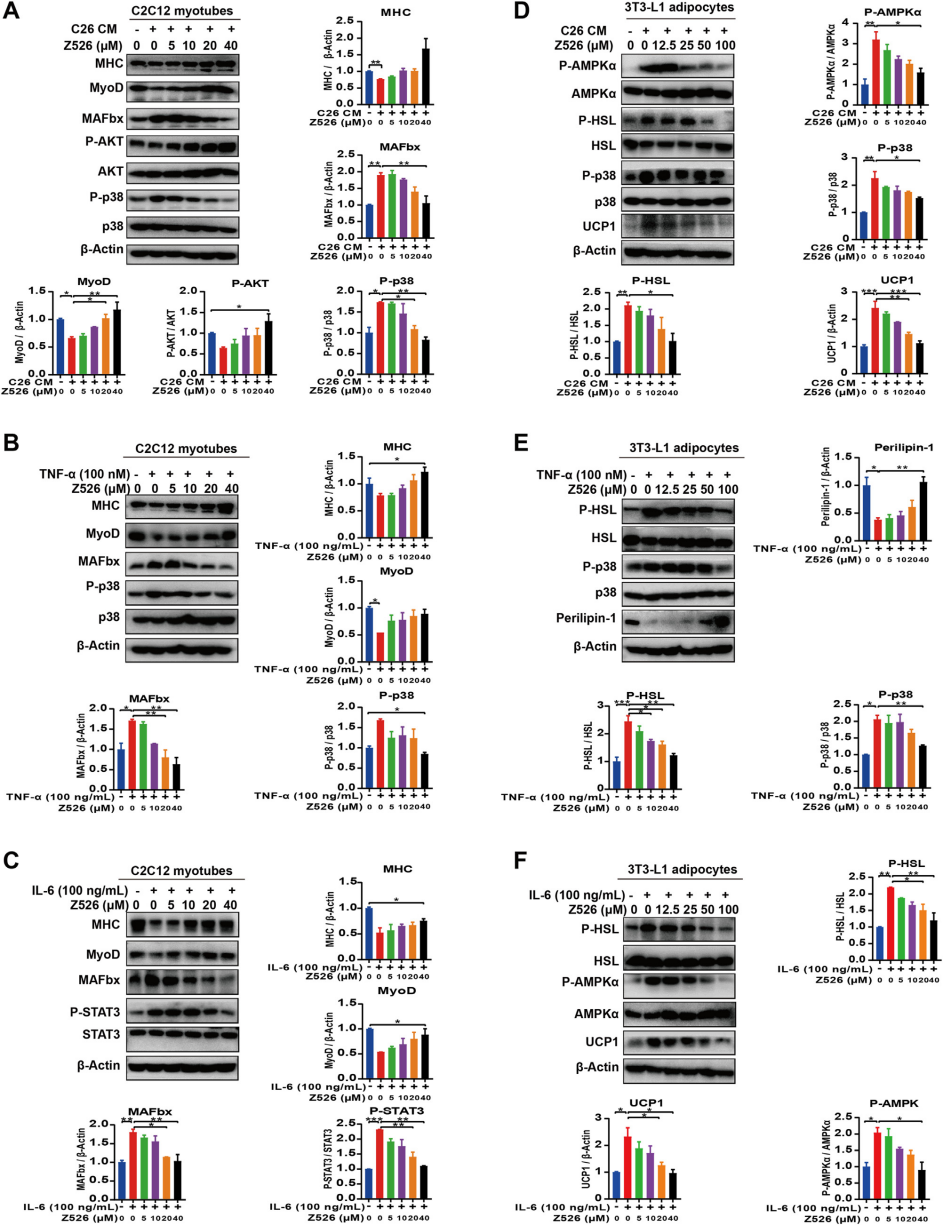
Z526 regulates metabolic signaling pathways in myotubes and adipocytes induced by cachectic injuries. **(A**–**C)** Western blotting analysis for signal proteins of C2C12 myotubes incubated with (A) C26 CM, (B) recombinant TNF-α, and (C) recombinant IL-6, in the presence or absence of Z526. **(D**–**F)** Western blotting analysis for signal proteins of 3T3-L1 adipocytes incubated with (D) C26 CM, (E) recombinant TNF-α, and (F) recombinant IL-6, in the presence or absence of Z526. The data represent mean ± standard error of the mean; ∗∗∗*P* < 0.001, ∗∗*P* < 0.01, ∗*P* < 0.05. CM, conditioned medium; IL-6, interleukin 6; TNF-α, tumor necrosis factor α.

To identify molecular mechanisms by which Z526 alleviates CAC, we detected associated NF-κB or ROS-mediated metabolic signaling pathways in C2C12 myotubes and 3T3-L1 adipocytes by western blotting assay. The results of signaling proteins involved in muscle atrophy in myotubes are shown in Figure 7A–C. C26 CM, TNF-α, or IL-6 induced a significant decrease in MHC and MyoD expression, which was indicative of myotube formation and differentiation. Z526 could reverse the decrease in a dose-dependent manner to ameliorate muscle atrophy. MAFbx, as a muscle-specific E3 ubiquitin ligase participating in protein degradation, showed a significant increase in C2C12 myotubes treated with C26 CM, TNF-α, or IL-6, which could be reversed by Z526. Additionally, Z526 alleviated the inhibition of AKT signaling, which controls protein synthesis in C26 CM-induced C2C12 myotubes. Either TNF-α-activated p38 or IL-6-activated STAT3 in C2C12 myotubes could enhance the activity of the ubiquitin-proteasome system, promoting protein degradation. The activation could be suppressed by Z526.

The results of key proteins participating in lipid loss in adipocytes are displayed in Figure 7D–F. C26 CM, TNF-α, or IL-6 increased phosphorylation of HSL, which accelerated lipolysis. Z526 could inhibit this activation in a dose-dependent manner to ameliorate lipolysis, while the mRNA and protein levels of HSL were not found to change (Fig. S4A). This activation could be reduced by Z526 dose-dependently. Uncoupling protein 1, a key component of the browning response, was observed to be elevated in 3T3-L1 adipocytes treated with C26 CM or IL-6, increasing energy expenditure, which could be reduced by Z526. Furthermore, either TNF-α-activated p38 or IL-6-activated AMPKα could enhance the activity of HSL, promoting lipid loss. Z526 could inhibit this activation. The elevated mRNA levels of zinc α2-glycoprotein in C26 CM-induced 3T3-L1 adipocytes, a fat-wasting marker, were reduced by Z526 (Fig. S4B).

**Figure 8:**
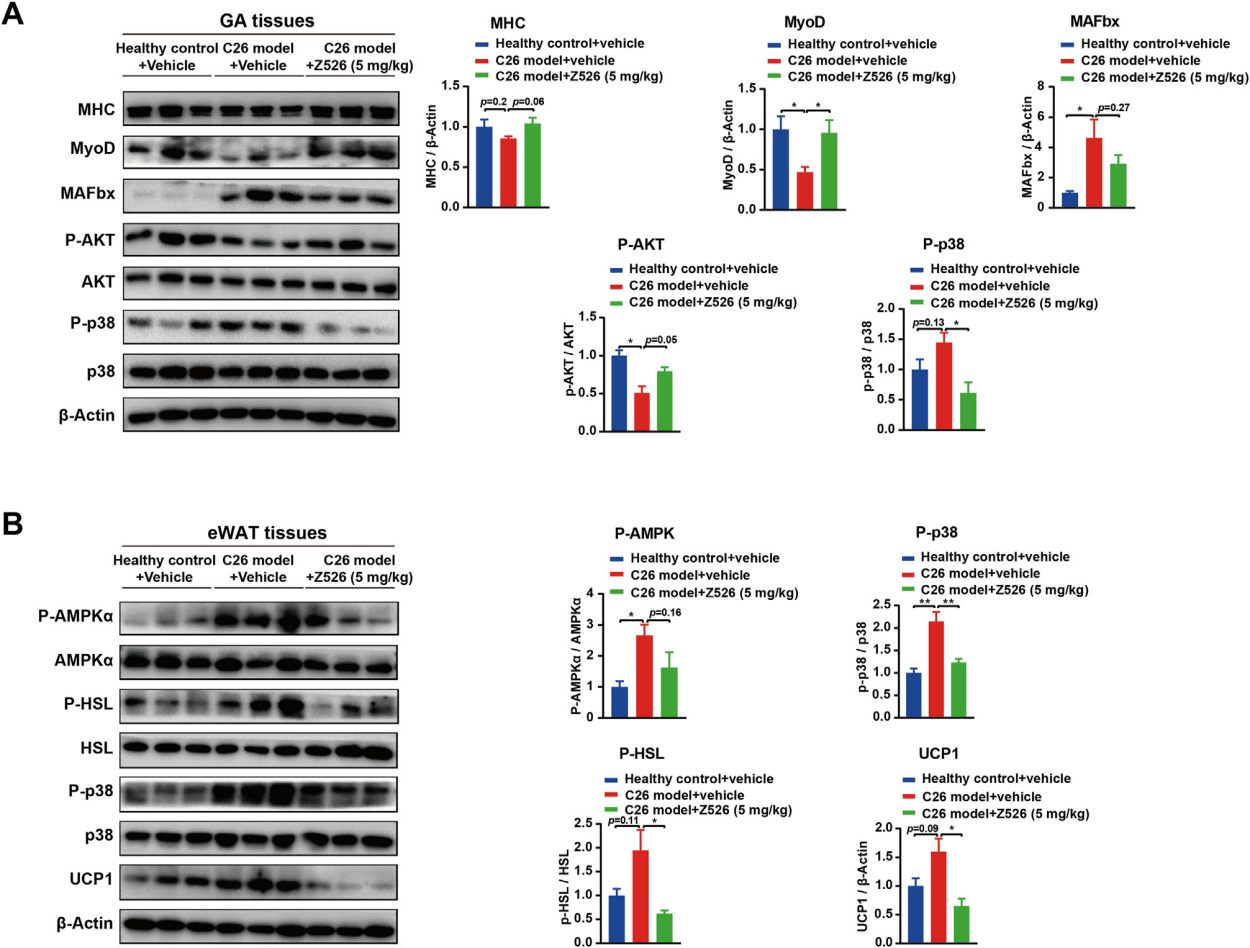
Z526 regulates metabolic signaling pathways in cachectic muscle and fat. After oral treatment of Z526, GA, and eWAT tissues were dissected from executed C26 tumor-bearing mice. **(A, B)** Western blotting analysis for signal proteins of GA tissues (A) and eWAT tissues (B). The data represent mean ± standard error of the mean; ∗∗∗*P* < 0.001, ∗∗*P* < 0.01, ∗*P* < 0.05. eWAT, epididymal white fat; GA, gastrocnemius muscle.

To validate these alterations in a mouse model, the signal proteins of GA and eWAT tissues harvested from C26 tumor-bearing mice were analyzed by western blotting assay. The functional proteins involved in protein synthesis (MHC, MyoD, AKT) were decreased or inhibited and the functional proteins involved in protein degradation (MAFbx, p38) were increased or activated in GA tissues of C26 tumor-bearing mice, and Z526 treatment reversed these changes (Fig. 8A). The functional proteins (AMPKα, HSL, p38) involved in lipolysis and uncoupling protein 1 involved in energy expenditure were overexpressed in eWAT tissues of C26 tumor-bearing mice, and were reversed by Z526 treatment (Fig. 8B). Overall, these results suggested that Z526 preserved the mass of muscle and fat by regulating the signaling pathways involved in metabolism.

### The preclinical safety assessment of Z526

**Figure 9:**
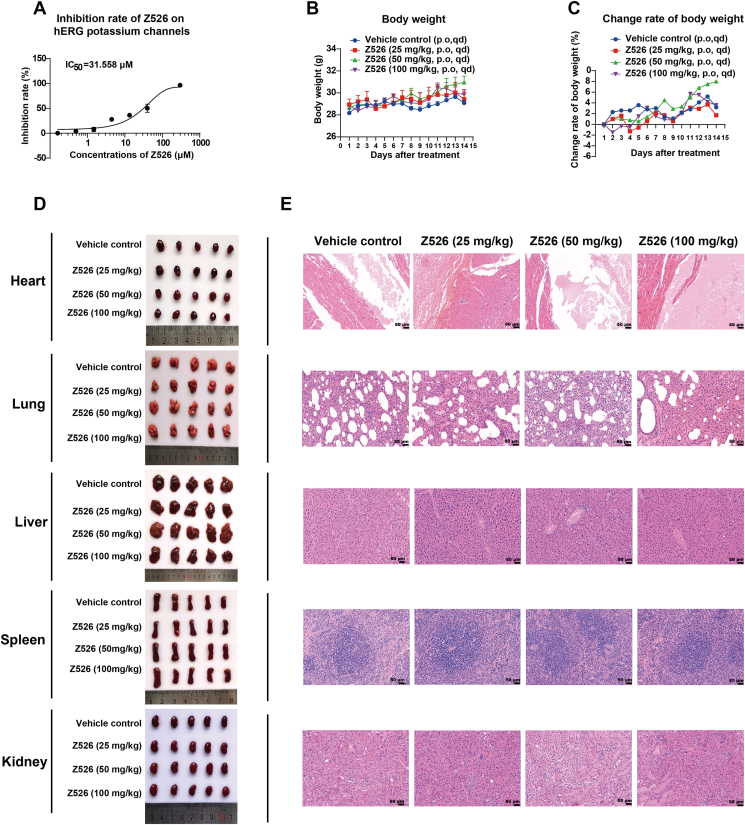
Evaluation of Z526's preclinical safety. ICR mice were orally administered with Z526 once daily, and their body weight and change rate of body weight were monitored. Paraffin sections of organs (heart, liver, spleen, lung, and kidney) dissected from executed mice after 14-day treatment were subjected to hematoxylin-eosin staining. **(A)** Z526's inhibitory effects on hERG potassium channels. **(B)** Body weight. **(C)** Change rate of body weight. **(D)** Representative images of mouse organs. **(E)** Representative images of tissue sections. Scale bar, 50 or 200 μm. The data represent mean ± standard error of the mean; *n* = 6.

Z526 did not inhibit human ether-a-go-go (hERG) related gene potassium channel (IC_50_ = 31.558 μM), suggesting a low risk for cardiotoxicity (Fig. 9A). The continuous administration of Z526 at various doses (25, 50, 100 mg/kg) did not affect ICR mice's body weight, which suggested that oral treatment of Z526 in mice at doses up to 100 mg/kg was well tolerated (Fig. 9B, C). Further histopathological examination of harvested major organ tissues (heart, lung, liver, spleen, and kidney) revealed no evidence of tissue toxicity after Z526 treatment at doses of 25, 50, and 100 mg/kg (Fig. 9D, E). The relative weights of tissues (tissue weight/body weight) are shown in Figure S5. These results indicated that Z526 had a favorable preclinical safety profile and was well tolerated at a high dose.

## Discussion

In recent years, CAC has attracted much more attention, on account of its contribution to poor quality of life and high mortality for cancer patients. So far, no therapeutic agent targeting CAC has been approved by the FDA in the clinic. In this study, we developed a novel anti-CAC candidate compound — Z526. Through systematic evaluation in multiple *in vitro* and *in vivo* models of CAC, Z526's efficacy of intervening in both NF-κB signaling and oxidative stress to combat CAC has been demonstrated.

In our previous study, we found that pyrrolidine dithiocarbamate exhibited potent anti-CAC activity.^24^ Nevertheless, it is not suitable for pyrrolidine dithiocarbamate to be developed into a new drug for the treatment of CAC, considering its poor membrane permeability, ineffective oral administration, neurotoxicity, high-dose administration, lack of intellectual property, *etc*.^24^, ^25^, ^26^ Based on the skeleton of dithiocarbamates, we have designed and synthesized a series of novel dithiocarbamate-like compounds. Through drug screening, Z526 was selected for a follow-up pharmacological study, owing to its favorable anti-CAC activity.

To comprehensively evaluate the anti-CAC efficacy of Z526, we used a variety of *in vitro* and *in vivo* models. *In vitro*, Z526 effectively alleviated C2C12 myotube atrophy and 3T3-L1 adipocyte lipolysis induced by CMs of multiple cachectic tumor cells or pro-cachectic inflammatory cytokines. *In vivo*, Z526 treatment attenuated cachectic symptoms of tumor-bearing mice by reducing muscle and fat loss and improving grip force via intraperitoneal or oral administration. Results of comparing the anti-CAC efficacy between Z526 and anamorelin (the only anti-CAC specific drug) showed that Z526 at 5 mg/kg displayed comparable anti-CAC efficacy to anamorelin at 30 mg/kg, and Z526 could preserve the grip force of mice better than anamorelin. Of note, Z526 significantly prolonged the survival time of LLC tumor-bearing mice. These studies fully demonstrated the anti-CAC potency of Z526.

Consistent with results in other studies,^16^^,^^19^^,^^27^ we also found activated NF-κB signaling and increased oxidative stress in cachectic muscle and fat of the CAC models. Our study supplied sufficient evidence that Z526 could intervene in NF-κB signaling and oxidative stress in cachectic muscle and fat. Since the pathogenic mechanisms of CAC are complex and multifactorial, a multiple-intervention therapeutic strategy may be more promising to achieve desired outcomes. NF-κB signaling and oxidative stress are extensively involved in metabolic signaling in muscle and fat. As the major resource of ROS, NADPH oxidases play roles in cancer-associated cachexia. Dasgupta et al found that NADPH oxidase 4, which was mediated by NF-κB, contributed to pancreatic cancer-induced cachexia.^28^ In our study, Z526 was found to reduce both mRNA and protein levels of NADPH oxidases in C26 CM-induced myotubes and adipocytes. Blockade of protein synthesis and increase in protein degradation, as shown by abnormal activation of the ubiquitin-proteasome system, are the main causes of muscle loss in CAC.^3^ AKT regulates protein synthesis and cell differentiation through the mTOR signaling pathway, while the phosphorylation of AKT is inhibited in the process of muscle atrophy, leading to down-regulation of MyoD and MHC (two markers of myofiber formation and differentiation) and arrested protein synthesis.^29^ Our study showed that Z526 could activate the AKT signaling pathway to increase the expression of MyoD and MHC in C2C12 myotubes and GA tissues. Since ROS is found to be able to suppress AKT via AMPK signaling,^30^ we speculated that Z526 could activate AKT signaling to promote protein synthesis by reducing excessive ROS. Moreover, activation of the ubiquitin-proteasome system is the main way of skeletal protein degradation. The activity of STAT3 and MAPK, which could be activated by ROS,^31^^,^^32^ were up-regulated in cachectic myotubes, triggering activation of the ubiquitin-proteasome system and resulting in protein degradation.^33^ This up-regulation of the ubiquitin-proteasome system could be decreased by Z526, characterized by a reduction of MAFbx, an E3 ubiquitin ligase involved in protein degradation. Similar results were also observed in muscle tissues of C26 tumor-bearing mice. Z526 might likely achieve this by reducing ROS-triggered activation. Mulder et al found that JNK signaling contributed to muscle wasting in pancreatic cancer cachexia and many studies also demonstrated that ROS could activate the JNK signaling pathway.^34^^,^^35^ During the progression of CAC, fat loss occurs earlier and faster than muscle. Our study found that Z526 also alleviated lipolysis of 3T3-L1 adipocytes, increased accumulative triglycerides in adipocytes, and decreased decomposed free glycerol. mRNA levels of zinc α2-glycoprotein, as a fat-wasting marker, were reversed in Z526-treated cachectic adipocytes. During adipocyte lipolysis, elevated cAMP in cells resulted in activation of AMP-dependent AMPKα, a mediator in energy metabolism.^36^ Activated AMPK signaling pathway elevated the phosphorylation of HSL, promoting lipolysis.^37^ Also, AMPK could up-regulate uncoupling protein 1 expression, facilitating the browning of white adipose and energy expenditure.^38^ AMPK was reported to be activated by ROS,^38^ which provided the possibility for Z526's regulation of lipolysis. In consistence with the results observed in myotubes, NF-κB and p38 MAPK in adipocytes were also excessively activated, leading to activation of HSL to facilitate lipolysis.^12^^,^^39^ Z526 was found to inhibit the activation of p38 MAPK to decrease the activity of HSL in 3T3-L1 induced by C26 CM or TNF-α, likely via reducing ROS signaling. Taken together, Z526 might regulate metabolic signaling pathways in muscle and fat to combat CAC via suppressing NF-κB and reducing ROS.

Evaluation of preclinical safety is essential for the development of drugs to treat CAC. In our findings, the hERG potassium channel blockade assay suggested that Z526 did not exhibit cardiotoxicity. Additionally, high doses of Z526 were well tolerated in mice. Further histopathological examination of harvested mouse major organ tissues also indicated that Z526 did not show tissue toxicity. Overall, Z526 has a favorable preclinical safety and is well tolerated at high doses.

The systemic inflammatory response, mediated by a signal network of cytokines, plays a dominant role in CAC, in which TNF-α and IL-6 are of particular concern.^39^ We found that Z526 treatment significantly reduced TNF-α and IL-6, suggesting that Z526 might be involved in the regulation of cachectic inflammation. Our further studies would focus on the effects of Z526 on the cachectic inflammatory response triggered by tumor cells and immune cells.

**Figure 10:**
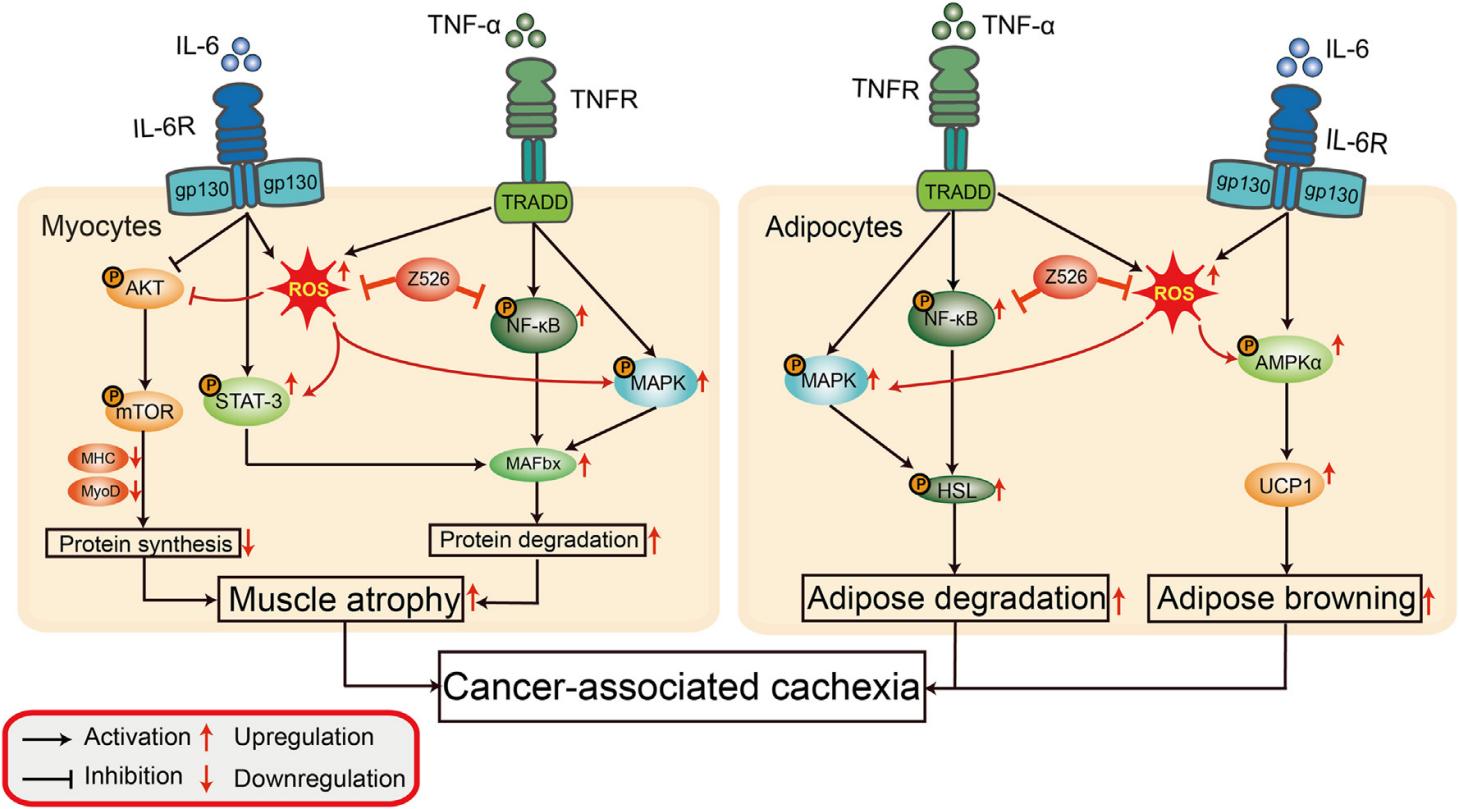
Schematic illustration of Z526's effects on cancer-associated cachexia. In cancer-associated cachexia, elevated TNF-α and IL-6 exacerbate muscle and fat loss by regulating multiple metabolic signaling pathways, which could be ameliorated by Z526's suppression of NF-κB signaling and oxidative stress. In cachectic muscle, Z526 alleviates muscle atrophy by promoting protein synthesis via AKT/mTOR signaling and reducing protein degradation via STAT-3/MAFbx, NF-κB/MAFbx, and MAPK/MAFbx; in cachectic fat, Z526 mitigates fat loss by suppressing MAPK/HSL and NF-κB/HSL to reduce adipose degradation and inhibiting AMPKα/UCP1 to decrease adipose browning. AKT, protein kinase B; AMPKα, catalytic alpha (α) subunit of serine/threonine kinase adenosine monophosphate-activated protein kinase; HSL, hormone-sensitive lipase; IL-6, interleukin 6; MAFbx, muscle atrophy F-box; MAPK, mitogen-activated protein kinase; mTOR, mammalian target of rapamycin; NF-κB, nuclear factor-kappa B; STAT-3, signal transducer and activator of transcription 3; TNF-α, tumor necrosis factor α; UCP1, uncoupling protein 1.

In this study, we identified Z526's potent anti-CAC effects in multiple experimental models of CAC *in vitro* and *in vivo* and its favorable preclinical safety profile. We also demonstrated that Z526 might regulate metabolic signaling in muscle and fat to combat CAC by intervening in multiple pathogenic mechanisms (NF-κB signaling and oxidative stress), as illustrated in Figure 10. Our study offers a promising candidate drug for CAC treatment and might also provide new insight into therapeutic strategies of multiple interventions.

## Ethics declaration

The study was approved by the Laboratory Animal Ethics Committee of East China Normal University (Approval Number: m20210106) and conducted in accordance with the Institutional Animal Care and Use Committee (IACUC) guidelines of East China Normal University.

## Author contributions

Xiaofan Gu performed most of the experiments, analyzed the data, and drafted the manuscript. Shanshan Lu assisted in the detection of protein levels in samples. Shanshan Lu, Yiwei Li, Meng Fan, and Yun Zhao assisted in animal experiments. Shuang Xu and Guangyu Lin synthesized the compounds. Yiyuan Liu assisted in taking fluorescent images. Xiongwen Zhang, Xiaochun Dong, Xuan Liu, and Weili Zhao contributed to the supervision of the project. Xiongwen Zhang, Xiaochun Dong, and Xuan Liu contributed to the design of the study and revision of the manuscript. All authors reviewed and approved the final manuscript.

## Conflict of interests

The authors declared no conflict of interests.

## Funding

This work was supported by the 10.13039/501100001809National Natural Science Foundation of China (No. 82373317, 82374085, 82303818, 22377087), the 10.13039/501100003399Science and Technology Commission of Shanghai Municipality, China (No. 20S11902200, 16DZ2280100), the 10.13039/100007219Natural Science Foundation of Shanghai Municipality, China (No. 23ZR1460500), and the 10.13039/501100002858China Postdoctoral Science Foundation (No. 2023M731106).
