# Supplementary material for: Novel oral compound Z526 mitigates cancer-associated cachexia via intervening NF-κB signaling and oxidative stress
Source: Genes Dis. 2024 Apr 8;12(2):101292. doi: 10.1016/j.gendis.2024.101292 (PMC11697116; doi:10.1016/j.gendis.2024.101292)
Supplement: Multimedia component 1 [file mmc1.docx]

**Supplementary materials**

Table S1 Reagents.

Table S2 Cell lines.

Table S3 Antibodies.

Table S4 Primer sequences.

Figure S1 The effect of Z526 on C2C12 myotube atrophy and 3T3-L1 adipocyte lipolysis in vitro.

Figure S2 The effect of Z526 on released free glycerol and intracellular triglycerides of 3T3-L1 adipocytes.

Figure S3 Z526 attenuates the cachectic symptoms of tumor-bearing mice in vivo.

Figure S4 RT-qPCR analysis for signal mRNAs of 3T3-L1 adipocytes.

Figure S5 Relative weight of tissues in Figure 9D.

**Table S1 Reagents**

| **Name** | **Manufacturer** |
| --- | --- |
| Z526, purity > 98% | Weili Zhao laboratories (Fudan University, China) |
| Recombinant murine IL-6 | PeproTech (Rocky Hill, NJ, USA) |
| Recombinant murine TNF-α | PeproTech (Rocky Hill, NJ, USA) |
| Mouse IL-6 ELISA Kit | MultiSciences (Hangzhou, China) |
| Mouse TNF-α ELISA Kit | MultiSciences (Hangzhou, China) |
| Reactive Oxygen Species Assay Kit | Beyotime (Shanghai, China) |

**Table S2 Cell line**

| **Name** | **Cell culture conditions** | **Source** |
| --- | --- | --- |
| C26 murine colon adenocarcinoma cells | The complete medium for the cell line is RPMI-1640 medium (containing 10% FBS and 1% P/S). Incubate the culture at 37℃ with 5% CO2 in air atmosphere. | Shanghai Institute of Materia Medica, Chinese Academy of Sciences |
| MC38 murine colon adenocarcinoma cells | The complete medium for the cell line is RPMI-1640 medium (containing 10% FBS and 1% P/S). Incubate the culture at 37℃ with 5% CO2 in air atmosphere. | ATCC, Manassas, USA |
| LLC murine lung adenocarcinoma cells | The complete medium for the cell line is DMEM (high-glucose), containing 10% FBS and 1% P/S. Incubate the culture at 37℃ with 5% CO2 in air atmosphere. | ATCC, Manassas, USA |
| HT-1080 human fibrosarcoma cells | The complete medium for the cell line is DMEM (high-glucose), containing 10% FBS and 1% P/S. Incubate the culture at 37℃ with 5% CO2 in air atmosphere. | ATCC, Manassas, USA |
| PANC-1 human pancreatic adenocarcinoma cells | The complete medium for the cell line is DMEM (high-glucose), containing 10% FBS and 1% P/S. Incubate the culture at 37℃ with 5% CO2 in air atmosphere. | ATCC, Manassas, USA |
| MIA PaCa-2 human pancreatic adenocarcinoma cells | The complete medium for the cell line is DMEM (high-glucose), containing 10% FBS and 1% P/S. Incubate the culture at 37℃ with 5% CO2 in air atmosphere. | ATCC, Manassas, USA |
| C2C12 murine myoblast | The complete medium for the cell line is DMEM (high-glucose), containing 10% FBS and 1% P/S. Incubate the culture at 37℃ with 5% CO2 in air atmosphere. | ATCC, Manassas, USA |
| 3T3-L1 murine pre-adipocytes | The complete medium for the cell line is DMEM (high-glucose), containing 10% FBS and 1% P/S. Incubate the culture at 37℃ with 5% CO2 in air atmosphere. | Shanghai Institute of Materia Medica, Chinese Academy of Sciences |

Notes: DMEM, Dulbecco’s Modified Eagle’s Medium. FBS, Fetal Bovine Serum. P/S, penicillin and streptomycin.

| **Name** | **Dilution** | **Cat No.** | **Manufacturer** |
| --- | --- | --- | --- |
| Mouse anti-β-Actin monoclonal antibody | 1:1000 | sc-8432 | Santa Cruz Biotechnology, Dallas, TX, USA |
| Mouse anti-MHC monoclonal antibody | 1:1000 | MF20 | DSHB, Iowa City, IA, USA |
| Rabbit anti-AKT monoclonal antibody | 1:1000 | 4691s | Cell Signaling Technology, Boston, USA |
| Rabbit anti-P-AKT monoclonal antibody | 1:1000 | 4060s | Cell Signaling Technology, Boston, USA |
| Rabbit anti-Atrogin-1 monoclonal antibody | 1:1000 | ab168372 | Abcam, MA, USA |
| mouse anti-STAT3 monoclonal antibody | 1:1000 | sc-8019 | Cell Signaling Technology, Boston, USA |
| mouse anti-P-STAT3 monoclonal antibody | 1:1000 | sc-8059 | Cell Signaling Technology, Boston, USA |
| Rabbit anti-p65 monoclonal antibody | 1:1000 | 8242s | Cell Signaling Technology, Boston, USA |
| Rabbit anti-P-p65 monoclonal antibody | 1:1000 | 3033s | Cell Signaling Technology, Boston, USA |
| Rabbit anti-p38 monoclonal antibody | 1:1000 | 8690s | Cell Signaling Technology, Boston, USA |
| Rabbit anti-P-p38 monoclonal antibody | 1:1000 | 4511s | Cell Signaling Technology, Boston, USA |
| Rabbit anti-AMPK monoclonal antibody | 1:1000 | 5832s | Cell Signaling Technology, Boston, USA |
| Rabbit anti-P-AMPK monoclonal antibody | 1:1000 | 2535s | Cell Signaling Technology, Boston, USA |
| Rabbit anti-Perilipin-1 monoclonal antibody | 1:1000 | #9349 | Cell Signaling Technology, Boston, USA |
| Rabbit anti-UCP1 monoclonal antibody | 1:1000 | #72298 | Cell Signaling Technology, Boston, USA |
| Rabbit anti-NOX2 polyclonal antibody | 1:1000 | 19013-1-AP | Proteintech, China |
| Rabbit anti-NOX4 polyclonal antibody | 1:1000 | 14347-1-AP | Proteintech, China |
| HRP-conjugated goat anti-mouse secondary antibody | 1:5000 | 70-GAM0072 | MultiSciences, Hangzhou, P.R. China |
| HRP-conjugated goat anti-rabbit secondary antibody | 1:5000 | 70-GAR0072 | MultiSciences, Hangzhou, P.R. China |

**Table S3 Antibodies**

**Table S4 Primer sequences**

| **Gene** | **Forward primer (5’-3’)** | **Reverse primer (5’-3’)** |
| --- | --- | --- |
| murine Nox2 | TGTGGTTGGGGCTGAATGTC | CTGAGAAAGGAGAGCAGATTTCG |
| murine Nox3 | CAACGCACAGGCTCAAATGG | CACTCTCGTTCAGAATCCAGC |
| murine Nox4 | TGCCTGCTCATTTGGCTGT | CCGGCACATAGGTAAAAGGATG |
| murine ZAG | GCCTTCTTCCACTACAACAG | TTCAGGACACTCCTCCTCTA |
| murine HSL | GCTGGAGGAGTGTTTTTTTGC | AGTTGAACCAAGCAGGTCACA |
| murine β-Actin | CTGTCCCTGTATGCCTCTG | ATGTCACGCACGATTTCC |


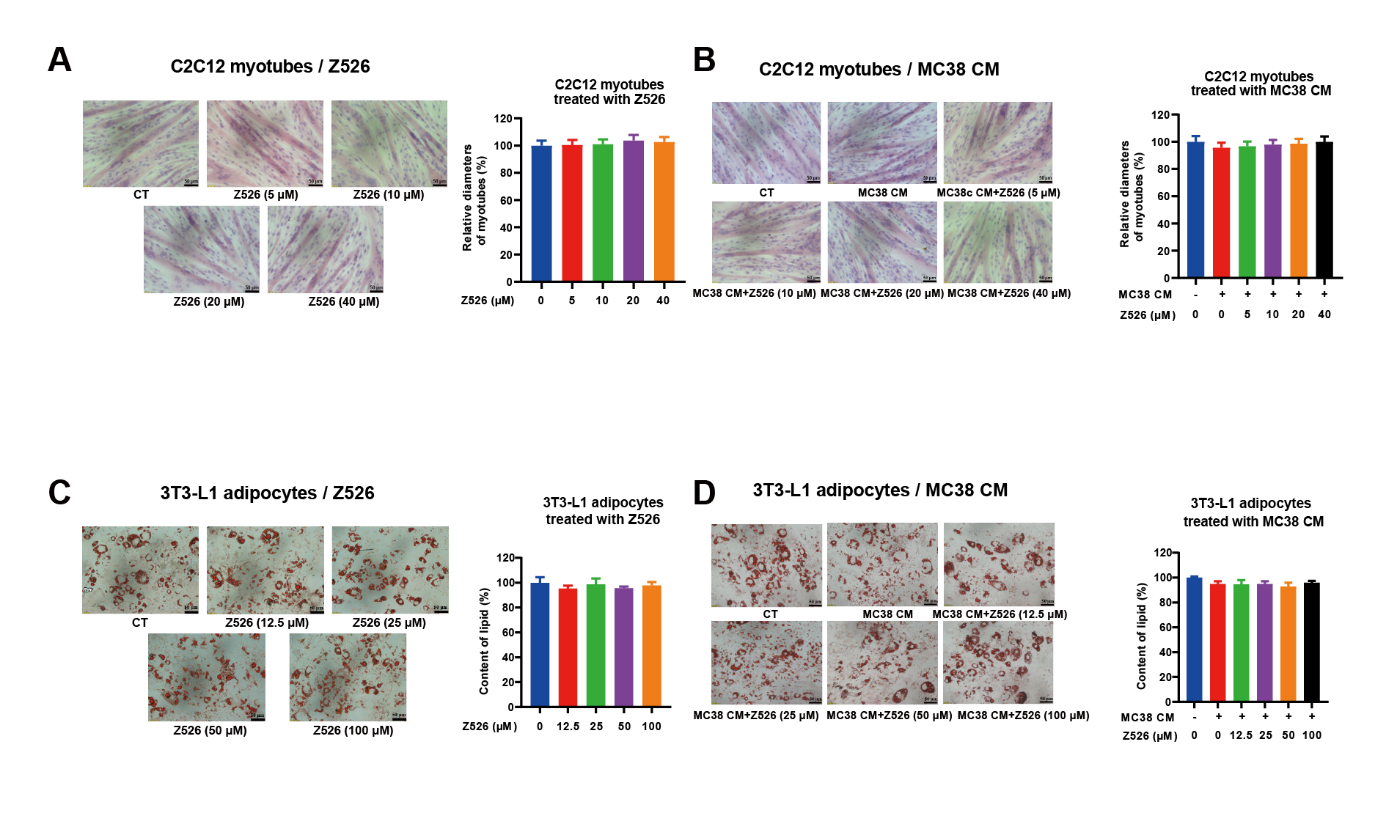


**Figure S1 The effect of Z526 on C2C12 myotube atrophy and 3T3-L1 adipocyte lipolysis *in vitro.*** (A) Representative images and quantitative statistics of diameters of C2C12 myotubes, treated with Z526. (B) Representative images and quantitative statistics of diameters of C2C12 myotubes treated with CM of MC38 tumor cells, in the presence or absence of Z526. (C) Representative images and quantitative statistics of lipid content of 3T3-L1 adipocytes, treated with Z526. (D) Representative images and quantitative statistics of lipid content of 3T3-L1 adipocytes treated with CM of MC38 tumor cells, in the presence or absence of Z526. Scale bar, 50 μm. The data represent the mean ± SEM.


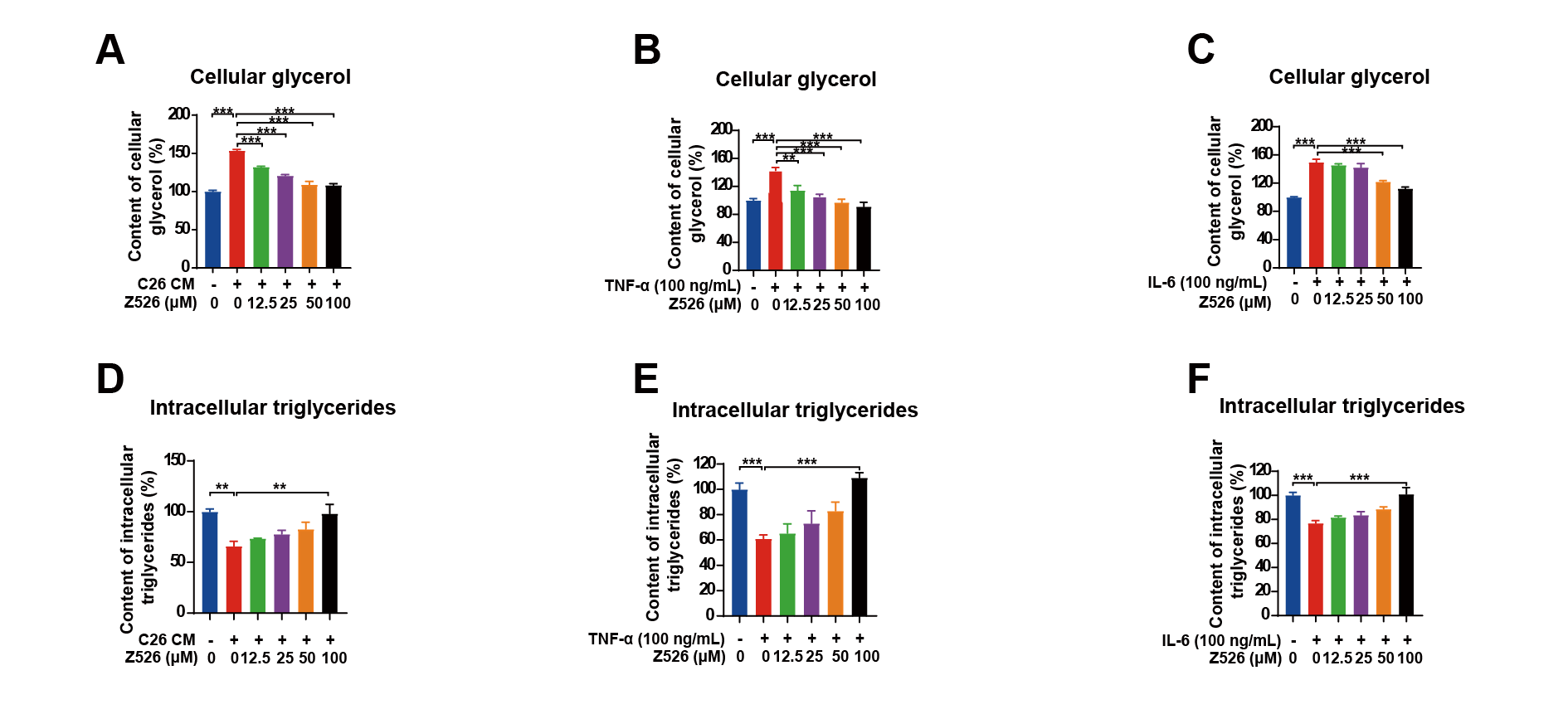


**Figure S2 The effect of Z526 on released free glycerol and intracellular triglycerides of 3T3-L1 adipocytes*.*** 3T3-L1 adipocytes were treated with C26 CM or pro-cachectic inflammatory cytokines in the presence or absence of Z526. Levels of released free glycerol of 3T3-L1 mature adipocytes treated with (A) C26 CM, (B) TNF-α and (C) IL-6. Levels of intracellular triglycerides of 3T3-L1 mature adipocytes treated with (D) C26 CM, (E) TNF-α and (F) IL-6. Scale bar, 50 μm. The data represent the mean ± SEM, ^***^*p*<0.001, ^**^*p* <0.01, ^*^*p* <0.05.


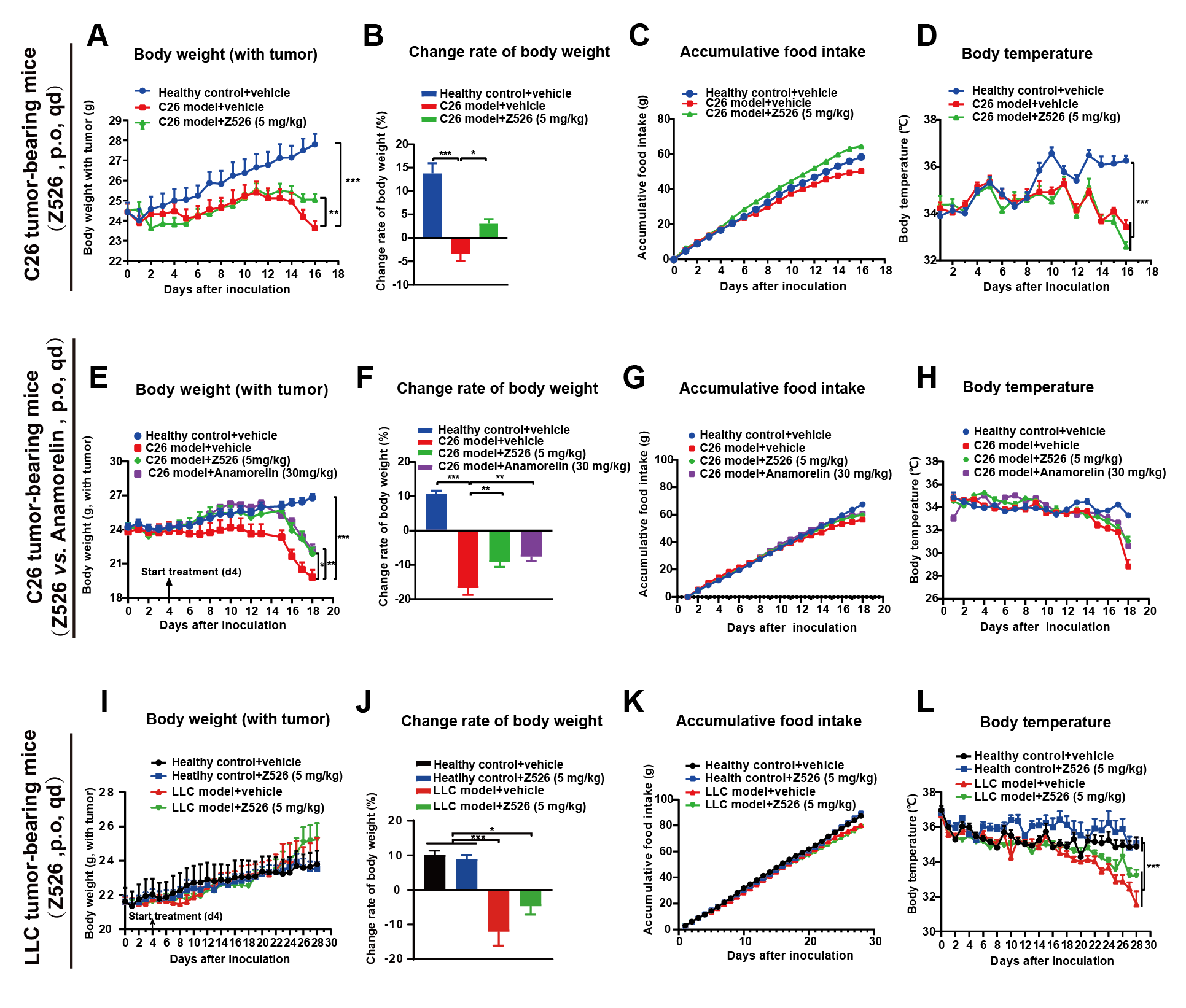


**Figure S3 Z526 attenuates the cachectic symptoms of tumor-bearing mice *in vivo*.** In C26 tumor-bearing mice, Z526 was administered orally and recorded parameters, including (A) Body weight with tumors, (B) Change rate of body weight at the last day, (C) Accumulative food intake and (D) Body temperature. In C26 tumor-bearing mice, Z526 or anamorelin was administered orally and recorded parameters, including (E) Body weight with tumors, (F) Change rate of body weight at the last day, (G) Accumulative food intake and (H) Body temperature. In LLC tumor-bearing mice, Z526 was administered orally and recorded parameters, including (I) Body weight with tumors, (J) Change rate of body weight at the last day, (K) Accumulative food intake and (L) Body temperature. The data represent the mean ± SEM, n=8, ^***^*p* <0.001, ^**^*p* <0.01, ^*^*p* <0.05.


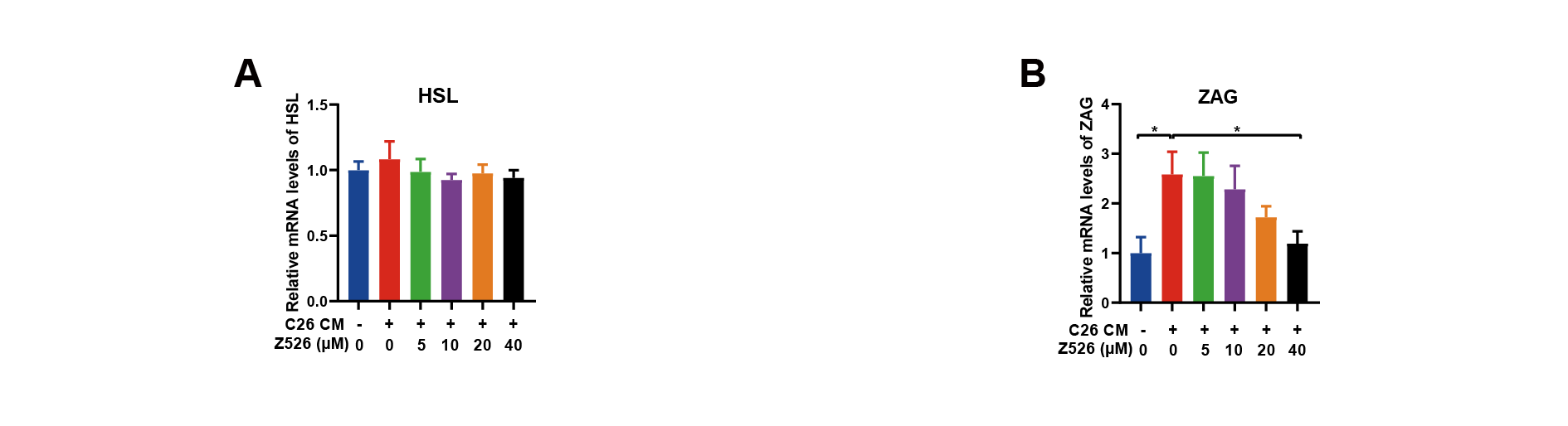


**Figure S4 RT-qPCR analysis for signal mRNAs of 3T3-L1 adipocytes.** 3T3-L1 adipocytes were treated with C26 CM, in the presence or absence of Z526. Quantitative statistics of mRNA levels of HSL (A) and ZAG (B) in 3T3-L1 adipocytes. The data represent the mean ± SEM, ^***^*p* <0.001, ^**^*p* <0.01, ^*^*p* <0.05.


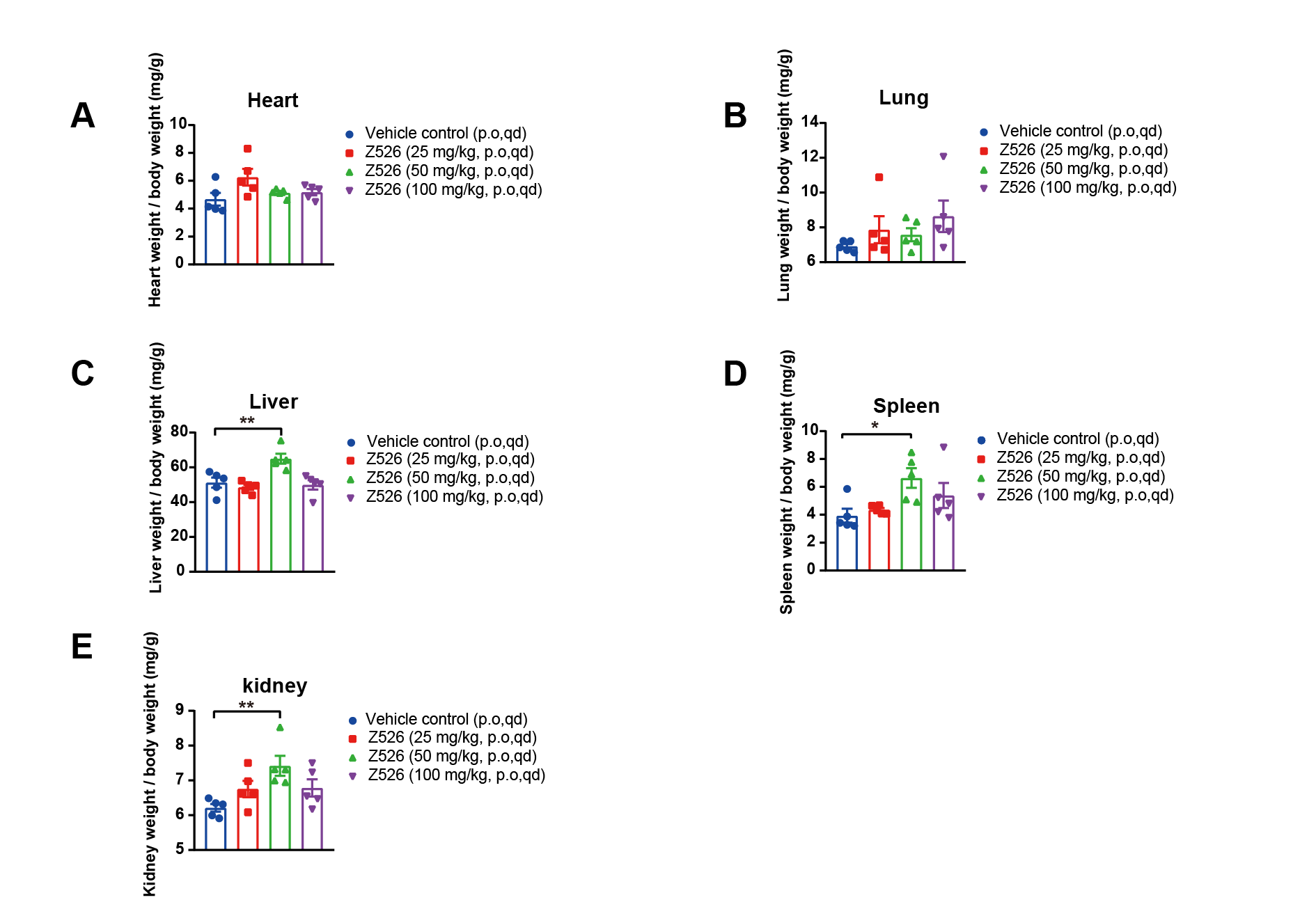


**Figure S5 Relative weight of tissues in Figure 9D.** ICR mice were daily administered with Z526 at various doses, and mice’s major tissues were dissected after 14-day treatment. Relative weight of (A) Heart, (B) Lung, (C) Liver, (D) Spleen and (E) Kidney. The data represent the mean ± SEM, n=6, ^***^*p*<0.001, ^**^*p*<0.01, ^*^*p*<0.05.
